# Activation of Glial FGFRs Is Essential in Glial Migration, Proliferation, and Survival and in Glia-Neuron Signaling during Olfactory System Development

**DOI:** 10.1371/journal.pone.0033828

**Published:** 2012-04-06

**Authors:** Nicholas J. Gibson, Leslie P. Tolbert, Lynne A. Oland

**Affiliations:** Department of Neuroscience, University of Arizona, Tucson, Arizona, United States of America; National Institutes of Health (NIH), United States of America

## Abstract

Development of the adult olfactory system of the moth *Manduca sexta* depends on reciprocal interactions between olfactory receptor neuron (ORN) axons growing in from the periphery and centrally-derived glial cells. Early-arriving ORN axons induce a subset of glial cells to proliferate and migrate to form an axon-sorting zone, in which later-arriving ORN axons will change their axonal neighbors and change their direction of outgrowth in order to travel with like axons to their target areas in the olfactory (antennal) lobe. These newly fasciculated axon bundles will terminate in protoglomeruli, the formation of which induces other glial cells to migrate to surround them. Glial cells do not migrate unless ORN axons are present, axons fail to fasciculate and target correctly without sufficient glial cells, and protoglomeruli are not maintained without a glial surround. We have shown previously that Epidermal Growth Factor receptors and the IgCAMs Neuroglian and Fasciclin II play a role in the ORN responses to glial cells. In the present work, we present evidence for the importance of glial Fibroblast Growth Factor receptors in glial migration, proliferation, and survival in this developing pathway. We also report changes in growth patterns of ORN axons and of the dendrites of olfactory (antennal lobe) neurons following blockade of glial FGFR activation that suggest that glial FGFR activation is important in reciprocal communication between neurons and glial cells.

## Introduction

**Figure 1:**
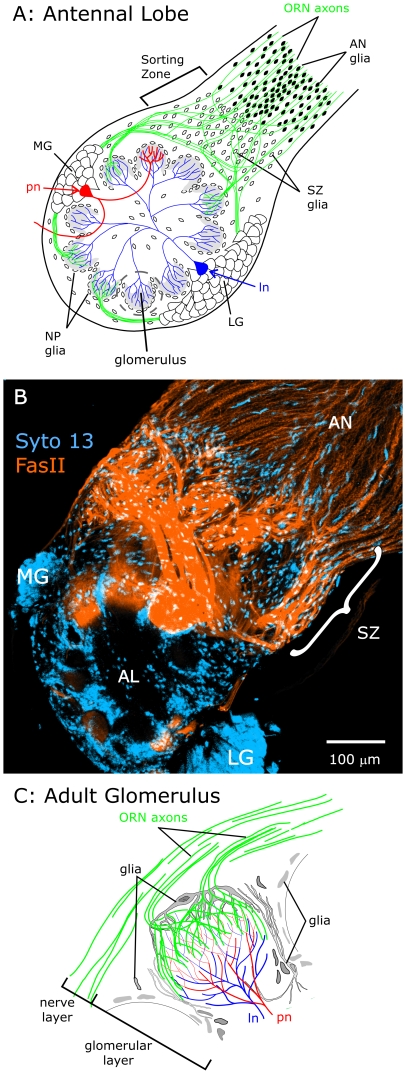
Diagram showing the basic cellular elements of an adult antennal lobe in *Manduca sexta*. **A:** Olfactory receptor neurons (ORNs) located in the antennae extend axons (green) to the antennal lobes of the brain where they end in structures called glomeruli and synapse with antennal lobe neurons. Two classes of AL neurons, local interneurons (ln) and projection neurons (pn), have their cell bodies in clusters called the lateral and medial groups (LG & MG), which reside outside of the antennal lobe neuropil. **B:** Labeling of an untreated female antennal lobe (AL) at stage 7 with an antibody to *M. sexta* Fasciclin II (orange) and a nucleic acid dye (Syto 13, blue) makes clear the major changes in ORN axon fasciculation and direction a short distance into the sorting zone (SZ), with axons exiting the sorting zone in large MFas II-positive bundles. Projection depth = 15 µm. **C:** A single glomerulus, showing the relationship of ORN axon terminals and AL neuron dendrites. ORN axons form a nerve layer around the outside of the antennal lobe neuropil, then turn sharply and extend through the glial layer and branch in the outer portion of a glomerulus in the glomerular layer. The cell bodies and processes of neuropil (NP) glial cells form a nearly complete envelope around each glomerulus. Panels A and C adapted from [33].

The past decade has seen a growing appreciation of the importance of neuron-glia signaling in nervous system development, and glial cells have been shown to play numerous roles affecting axon outgrowth or growth arrest, course changes, fasciculation, and targeting [1]–[10]. In the experimentally advantageous developing primary olfactory system of the adult moth, *Manduca sexta*, several interactions between neurons and glia have been well characterized [5]. Olfactory receptor neurons (ORNs) send their axons in the antennal nerve (AN) toward the nascent adult antennal lobe of the brain where the first axons to arrive induce a change in a subset of central glial cells, causing them to proliferate and migrate outward a short distance into the nerve (Fig. 1A). These glial cells then define an axonal sorting zone (SZ); their presence induces subsequently arriving ORN axons to change course and fasciculate with other ORN axons with which they then travel to a given region of the antennal lobe (AL) (Fig. 1B). The terminal branches of ORN axons form protoglomeruli on which the array of mature glomeruli is built. The ORN axons eventually form synapses with dendrites of antennal lobe neurons (Fig. 1C). Formation of the protoglomeruli induces the remaining antennal-lobe glial cells (termed neuropil-associated or NP glia) to migrate to surround and stabilize the developing glomerular structures [11], [12]. In glia-deficient animals or animals in which drug treatment blocks glial cell migration and process extension, the glomerular organization disintegrates [12]–[15]. In addition, glial deficiency in the sorting zone causes defects in axon fasciculation and targeting [16].

In previous studies, we identified several molecular signals that could underlie these neuron-glia interactions in the primary olfactory pathway of *M. sexta*. The transmembrane form of *M. sexta* Fasciclin II (TM-MFas II, an immunoglobulin-superfamily cell adhesion molecule (IgCAM) and a homolog of vertebrate NCAM) is found on a subset of ORN axons and the GPI-linked form of *M. sexta* Fasciclin II (GPI-MFas II) is expressed by antennal nerve (AN) glial cells and in the perineurial sheath [17]. Neuroglian (also an IgCAM and a homolog of vertebrate L1) is expressed on ORN axons and on NP and SZ glia ([18]; Oland, unpublished), and Epidermal Growth Factor Receptors (EGFRs) are found on ORN axons [18]. EGFRs were found to be phosphorylated (indicative of activation) only on ORN axons in the sorting zone and protoglomeruli, suggesting that activation depended on interactions with, or proximity to, NP and SZ glia. Blocking EGFRs caused ORN axon stalling and loss of axon fasciculation in the sorting zone [18].

In this paper, we pursue evidence that suggests roles for the Fibroblast Growth Factor Receptors (FGFRs), which are present on glial cells during critical stages of development [18]. FGFRs represent an additional possible signaling partner linking glia and axons reciprocally via Neuroglian and MFasII. Work by several groups has shown that homophilic interactions (in *cis* and in *trans*) between IgCAMs can lead to activation of both EGFRs and FGFRs with subsequent effects on direction and degree of neuron migration and axon extension [19]–[23]. In the current study, we find that FGFRs are present and activated (phosphorylated on kinase-domain tyrosines) on SZ, NP, and AN glia during developmental stages important in axon ingrowth and sorting and in the formation of olfactory glomeruli in the antennal lobe. Pharmacologic blockade of FGFR activation leads to the absence of migration by NP, but not SZ or AN, glial cells. Blockade of glial FGFRs also leads to aberrant ORN axon outgrowth. Because we find no evidence for FGFRs on ORNs, this suggests that activation of glial FGFRs is important in glia-to-ORN signaling. As it does in many other systems [24], [25], FGFR activation also appears to be essential for glial cell survival, as blockade leads to widespread glial cell loss at later stages.

## Materials and Methods

### Animals

*Manduca sexta* (Lepidoptera: Sphingidae) were reared from eggs on an artificial diet in a laboratory colony essentially as described by Sanes and Hildebrand [26]. The adult antennal system develops during metamorphosis, when the animal changes from larva to moth. This phase can be divided into 18 stages, each lasting 1–4 days. Animals were staged according to features, such as eye pigmentation and leg development, visible through the cuticle under fiber-optic illumination as described by Tolbert et al. [27] and Oland and Tolbert [11].

### Removal of antennal input

In some animals, the antennal lobe on one side was deprived of ORN axon input throughout development, using surgical methods described previously [11], [18]. Briefly, animals at stage 1 of adult development were anaesthetized by exposure to CO_2_. The cuticle covering the base of one antenna was removed and the underlying part of the antennal anlage removed with forceps. The opening was then filled with melted wax to prevent ORN axons from surviving distal receptor neurons from extending toward the brain, and the animals were returned to the rearing facility and allowed to develop under standard conditions. Because ORN axons do not project contralaterally, the antennal lobe on the operated side received no input from ORNs. The antenna on the opposite (control) side was not disturbed and therefore received normal afferent input.

### Primary antibodies for immunocytochemistry

When possible, antibodies developed against *Manduca sexta* proteins were used. Alternatively, antibodies developed against proteins from vertebrate species were used if the antigenic sequence was a close match to the corresponding amino acid sequence of *Manduca* or of *Bombyx mori*, which we have found to exhibit very little sequence difference from *Manduca*.

#### Manduca Fasciclin II

Mouse monoclonal antibody P1E1-1C3 (“C3”), developed against the extracellular domain common to all isoforms of *Manduca sexta* Fasciclin II (MFas II) [17], [28] was the generous gift of Dr. Philip Copenhaver, Oregon Health Sciences University, Portland, OR.

#### FGFR

We used a polyclonal antibody to activated human FGFR1 (phosphorylated on tyrosines 653 and 654) which was developed against a phospho-peptide having amino acid identity between human and *Bombyx mori* in 8 of 11 amino acids (#3471, Cell Signaling Technology, Danvers, MA). The antigenic phospho-peptide was used for preadsorption assays. We also used an antibody to the extracellular domain of human FGFR1 (also known as Flag, #05-149, Upstate Biotechnology, Lake Placid, NY) and an antibody to heparan sulfate (#375080, Calbiochem, San Diego, CA) because heparan sulfate proteoglycans are necessary co-receptors for FGF and are expected to colocalize with the FGFR [29]-[31].

#### EGFR

An antibody to activated human EGFR (phosphorylated at tyrosine residue 845; #2231, Cell Signaling Technology, Beverly, MA) was chosen based on sequence homology with the corresponding region of EGFR of *Bombyx mori and Manduca sexta*. The antibody specificity has been checked with blocking peptides and western blots, and the distribution of activated EGFRs in the moth olfactory pathway during development has been described [18], [32].

#### Ankyrin B

A mouse monoclonal antibody generated against a peptide corresponding to the spectrin-binding domain of human Ankyrin B was purchased from Zymed Laboratories ((#33-3700, Invitrogen). In *Manduca* antennal lobes, this antibody recognizes a subset of ORN axons that terminate in a single glomerulus located dorso-posteriorly in the antennal lobe [33]. It is used here as a marker for this axonal subset (not as a means of monitoring ankyrin B expression).

#### Phospho-histone H3

An affinity-purified rabbit polyclonal antibody (#H0412) developed against a phospho-peptide corresponding to amino acids 7–20 (pSer10) of human histone H3 was purchased from Sigma, St Louis, MO. Histone-H3 in humans and *Bombyx* exhibit 100% amino acid identity.

### Immunocytochemistry

Animals at various stages of metamorphic adult development were anesthetized by cooling on ice. Brains were dissected under insect saline solution (150 mM NaCl, 4 mM KCl, 6 mM CaCl_2_, 10 mM HEPES, 5 mM glucose, pH 7.0, adjusted to 360 mOsm with mannitol; [34]. The perineurial sheath covering the brain was removed to aid in fixative and antibody penetration. All tissue, except as noted, was vibratome-sectioned (Vibratome, Technical Products International, St. Louis, MO) at 100 µm. The final step in all protocols, also unless noted, was clearing the brains or sections for 15 min each first in 50% glycerol in water, then in 80% glycerol in water, and finally mounting on slides in 80% glycerol. For some preparations, glial cell nuclei also were labeled with the nucleic acid stains Syto 13 or Syto 59 (Molecular Probes, #S-7575 and #S-11341). Sections were washed in 10 mM Tris (pH 7.4), then incubated in the Syto dye 1∶10,000 in Tris (not phosphate buffer) for 60 min, washed in Tris, and mounted in H_2_O/glycerol. Glial nuclei were identified by their small size compared to neuronal nuclei, and by their location either in the axonal sorting zone region of the antennal nerve where they are the only cell type present [16] or in the envelope surrounding each glomerulus [11], [35].

Specific protocols for each antibody appear in Table 1. For each antibody, some brains were processed as shown but without addition of primary antibodies to control for nonspecific immunolabeling. In addition, a pre-adsorption control was carried out for the pFGFR antibody. Two µl of the anti-pFGFR antibody and 20 µl of the antigenic phosphopeptide were added to 5 µl TBSA containing 10% BSA and mixed on a rotator at RT for 1 hr. The mixture was then used in immunocytochemistry of two sectioned brains prepared as described above. Additional brains were labeled with the pFGFR antibody and with the secondary antibody alone as controls for methods of processing and for nonspecific labeling.

**Table 1:** Immunocytochemistry Protocols.

antibody	fixative solution	postfix	block	primary	wash	secondary
Fibroblast growth factor receptor, FGFR (mouse)	M/F (9∶1) on ice; ON, −20°C	No	0.1% T, 0.1% A, 2% BSA in TBS; 1 hr, RT	2 ul in 500 ul in block; ON, RT	0.1% T in TBS	2 ul Cy3 Goat anti-mouse IgG+IgM in 500 ul block; ON, RT
HSPG (mouse)	M/F (9∶1) on ice; ON, −20°C	No	0.1% A, 2% BSA in PBS; 1 hr, RT	1 ul in 500 ul in block; ON, RT	0.1% T in PBS	2 ul Cy3 Goat anti-mouse IgG+IgM in 500 ul block; ON, RT
Activated FGFR, pFGFR (rabbit)	M/F (9∶1) on ice; ON, −20°C	No	0.1% T, 0.1% A, 2% BSA in TBS; 1 hr, RT	1 ul in 500 ul block; ON, RT	0.1% T in TBS	2 ul Cy3 Goat anti-rabbit in 500 ul block; ON, RT
Activated Epidermal growth factor receptor, pEGFR (rabbit)	2.5% P, 1% G, 1% SMB, in 0.1 M cacodylate buffer, pH 7.2; ON, 4°C after microwave	NaBH_4_	0.1% T, 0.1% A, 2% BSA in TBS; 1 hr, RT	1 ul in 500 ul block; ON, RT	0.1% T in TBS	2 ul Cy3 Goat anti-rabbit in 500 ul block; ON, RT
Manduca Fasciclin II, C3 (mouse)	4% P or 4%P, 0.1% G, in 0.1 M PB	No	0.5% T, 0.1% A, 2% BSA in TBS; 1 hr, RT	1∶10,000 in 500 ul block; ON, RT	0.1% T in TBS	2 ul Cy3 Goat anti-mouse IgG+IgM in 500 ul block; ON, RT
Phospho-histone H3 (rabbit)	4% P in 0.1 M PB; ON, 4°C	DNAse	0.5% T, 0.1% A, 2% NGS in PBS; 1 hr, RT	1.5 ul in 500 ul block; ON, RT	0.1% T in PBS	2.5 ul Cy3 Goat anti-rabbit F(ab)_2_ in 500 ul PBS with 0.1% A, 0.1% T; 3–4 hrs, RT
Ankyrin B (mouse)	2.5% P, 1% G, 1% SMB in 50 mM carbonate buffer (pH 9.4), final pH 10.8; ON, 4°C after microwave	NaBH_4_	0.1% T, 0.1% A, 2% BSA in TBS; 1 hr, RT	2 ul in 500 ul block; ON, RT	0.1% T in TBS	2 ul Cy3 Goat anti-mouse IgG+IgM in 500 ul block; ON, RT, then 2 ul Alexa 564-Donkey anti-goat in 500 ul block, ON, RT

Microwave protocol: 18°C; Power level 2; 2 min on/2 min off/2 min on/2 min off.

Pella research-grade oven (# 3450, with variable power controller #3430 and cold spot).

A: sodium azide.

Alexa 564-Donkey anti-goat tertiary (Molecular Probes #A11056).

BSA: bovine serum albumin, IgG-free (Jackson Immunoresearch #001-000-161).

Cy3-Goat anti-mouse secondary (Jackson #115-165-068).

Cy3-Goat anti-rabbit secondary (Jackson # 111-165-144).

Cy3-Goat anti-rabbit F(ab)_2_ (Jackson # 111-166-047).

DNAse I (Sigma): 10 U DNAse, 4 mM MgCl2 in PBS; 1 hr, 37°C.

G: glutaraldehyde.

M/F: methanol/37% formalin.

NaBH_4_: 0.01 M NaBH_4_, 0.5% SMB in 0.05 M Tris HCl, pH 7.5; 30 min.

NGS: normal goal serum.

ON: overnight on a shaker.

P: paraformaldehyde.

PB: phospate buffer pH 7.4.

PBS: phosphate-buffered saline (10 mM sodium phosphate, 150 mM NaCl, pH 7.4).

RT: room temperature.

SMB: sodium metabisulfite.

T: Triton X-100.

TB: 10 mM Tris-HCl, pH 7.4.

TBS: Tris-buffered saline (20 mM Tris-HCl, 150 mM NaCl, pH 7.4).

### Lectin labeling

Brains that had been fixed on a shaker ON at 4°C in 4% paraformaldehyde plus 0.1% glutaraldehyde in 0.1 M phosphate buffer, pH 7.4, were sectioned and incubated ON at 4°C in 0.5 ml lectin buffer (300 mM NaCl, 100 µM CaCl2 in 10 mM HEPES, pH 7.5) containing 2 µl (10 µg) of fluorescein-labeled *Artocarpus integrifolia* lectin (Jacalin) or *Lycopersicon esculentum* lectin (LEL) (Vector Laboratories, Burlingame, CA).

### Inhibition of FGFR activity

The highly selective, cell-permeable FGFR inhibitor PD173074 was the generous gift of Pfizer, Inc. Additional drug was purchased from Sigma (#P2499) and from Tocris Bioscience (#3044, Ellisville, MO). It has been used to block activation of FGFRs in vertebrates [36]–[38] and in *Drosophila* [39]. Because relatively few gene sequences are known for *Manduca sexta*, and because our Clustal-W amino acid alignments have shown a high degree of identity between *Bombyx* and known *Manduca* proteins, we used the sequence for the published *Bombyx mori* FGFR [40] to ask if the amino acids that contact PD173074 in the highly conserved ATP binding pocket of the human FGFR1 [36] were also present in *Bombyx*. An amino acid alignment of human FGFR1 and the *Bombyx* FGFR showed that the *Bombyx* sequence matches the human sequence at all the contact sites (Fig. S1). Thus there was a high probability that PD173074 would work in an effective and selective manner in *Manduca* as it has in both vertebrates and *Drosophila*. Animals at early stage 3 were anaesthetized by incubation in CO_2_ for 20 min. PD173074 (dissolved in DMSO at 0.5 mg/10 µl = 0.1 M, n = 84) or DMSO alone (n = 31) was injected into the headspace at various stages of adult development. The injection sites were sealed with melted dental wax and the animals returned to the rearing room to continue development. Early results suggested that higher single doses sometimes produced less effect than lower single doses, possibly indicating that at high doses the drug, which is dissolved in DMSO, precipitated out as it was injected into the aqueous hemolymph. For this reason, and because we were concerned that newly expressed FGFRs might overwhelm single drug injections, two or three 0.5 mg injections spaced 24 hr apart were used rather than a single, larger injection. We found no difference in phenotype between animals receiving 2 vs 3 injections.

### Labeling of dividing cells

Four control (DMSO-injected) and six experimental (0.5 mg PD173074 in DMSO) animals at stage 3 of development were divided into 1× and 2× (24 hr apart) injection groups. The 1× animals were injected with the drug or vehicle only on the 2^nd^ day, and all animals were dissected on the fifth day after injection, so that we could observe animals at two doses and at two developmental stages, mid 5 and late5-early 6. Brains were fixed, sectioned at 100 µm, and processed for phospho-histone H3 immunocytochemistry (a marker of cells undergoing mitosis) as described in Table 1.

### Labeling of apoptotic cells

PD173074-treated (n = 9) and control (n = 7) animals were assayed for apoptotic cells using the ApopTag Plus Fluorescein *In Situ* Apoptosis Detection Kit (#S7111, Chemicon International), which uses the TUNEL (terminal deoxynucleotidyl transferase dUTP nick end labeling) technique. Brains were fixed in 4% paraformaldehyde in 0.1 M phosphate buffer, pH 7.4, for 48 hr at 4°C. Fixed brains were washed in PBS, cryoprotected in 10, 20, and 30% sucrose in 0.1 M phosphate buffer, pH 7.4, at 4°C, flash-frozen in liquid propane, and cryosectioned at 20 µm. Sections were then processed according to the apoptosis detection kit instructions, using propidium iodide to counterstain nuclei.

### Western blot

Antennal lobes (with attached intracranial portion of antennal nerves) of three female animals at stage 7 of adult development were removed and solubilized in Novex lithium dodecyl sulfate sample buffer (InVitrogen, Carlsbad, CA) containing protease-inhibitor and phosphatase-inhibitor cocktails (#P2714 and P5726, Sigma, St Louis, MO). Solubilized samples were run on a Novex NuPAGE 4–12% Bis-Tris gel and transferred to a PVDF membrane as described previously [18]. Blots were incubated for 1 hr at RT in blocking solution (TBS + 0.1% Tween 20+5% BSA), then ON at 4°C in blocking solution with anti-pFGFR antibody (3 µl in 5 ml). Blots were then washed in TBS-Tween and incubated 4 hr at RT in blocking solution plus horseradish peroxidase-conjugated goat anti-rabbit antibody (Jackson Immunoresearch). Blots were washed again and developed using the Opti-4CN kit (Bio-Rad, Hercules, CA).

An additional blot to compare pFGFR labeling of antennal lobes treated with DMSO or PD173074 (2 daily injections beginning at stage mid-5, dissected at stage early-6) was done as described above using lobes and attached nerves from two animals for each treatment. Because immunocytochemistry suggested residual labeling in AL neuron cell bodies following PD173074 treatment, these cell body clusters were removed from the tissue prior to processing in order to assess solely the effect on glia.

### Confocal microscopy and image processing

Sections were viewed on a Nikon PCM 2000 or a Zeiss 510 Meta laser scanning confocal system (equipped with argon, green HeNe, and red HeNe lasers) using Simple 32 software (Compix Inc., Cranberry Township, PA) or LSM software (Zeiss), respectively. Optical sections were acquired at 1- to 5-µm intervals (depending on the objective used) through the depth of the antennal lobe and saved as three-dimensional stacks. To examine the localization of FGFRs, HSPGs, and Syto dyes to cellular sub-compartments, we used a 40×, oil immersion EC PLAN-NEOFLUAR, N.A. 1.3 lens (Zeiss). Vehicle controls were always imaged along with experimental brains and imaging parameters were always held constant when comparing between controls and experimental brains or across developmental stages. Confocal image stacks were projected and merged in false color using Confocal Assistant (copyrighted by Todd Brelje, distributed by Bio-Rad, Richmond, CA) or the Zeiss LSM image browser, and then imported into Corel Photopaint, where image hue, intensity, and contrast were adjusted for maximum clarity. The images were then combined into figures in Corel Draw, where annotations were added.

## Results

The primary olfactory pathway of *M. sexta* consists of a small number of cell types: a) ORNs, whose cell bodies are located distally in the antennae and whose axons extend to form the antennal nerves and antennal lobes, b) antennal-lobe neurons, whose cell bodies are clustered (primarily in two groups, lateral and medial) entirely outside of the antennal lobe neuropil and whose dendrites form synapses with each other and with the terminal arborizations of ORN axons in the glomeruli, c) centrally-derived glial cells which populate the sorting zone (SZ glia) and which surround the glomeruli (NP glia), and d) peripheral glia (antennal nerve (AN) glia), which migrate toward the antennal lobes along the antennal nerves and extend processes that surround small groups of ORN axons [16]. AL neuron cell bodies are much larger than those of glial cells (Fig. 1A,B), and they lie outside of the neuropil and its glial border, making labeling with neuronal or glial markers unnecessary for identification [11], [16].

**Figure 2:**
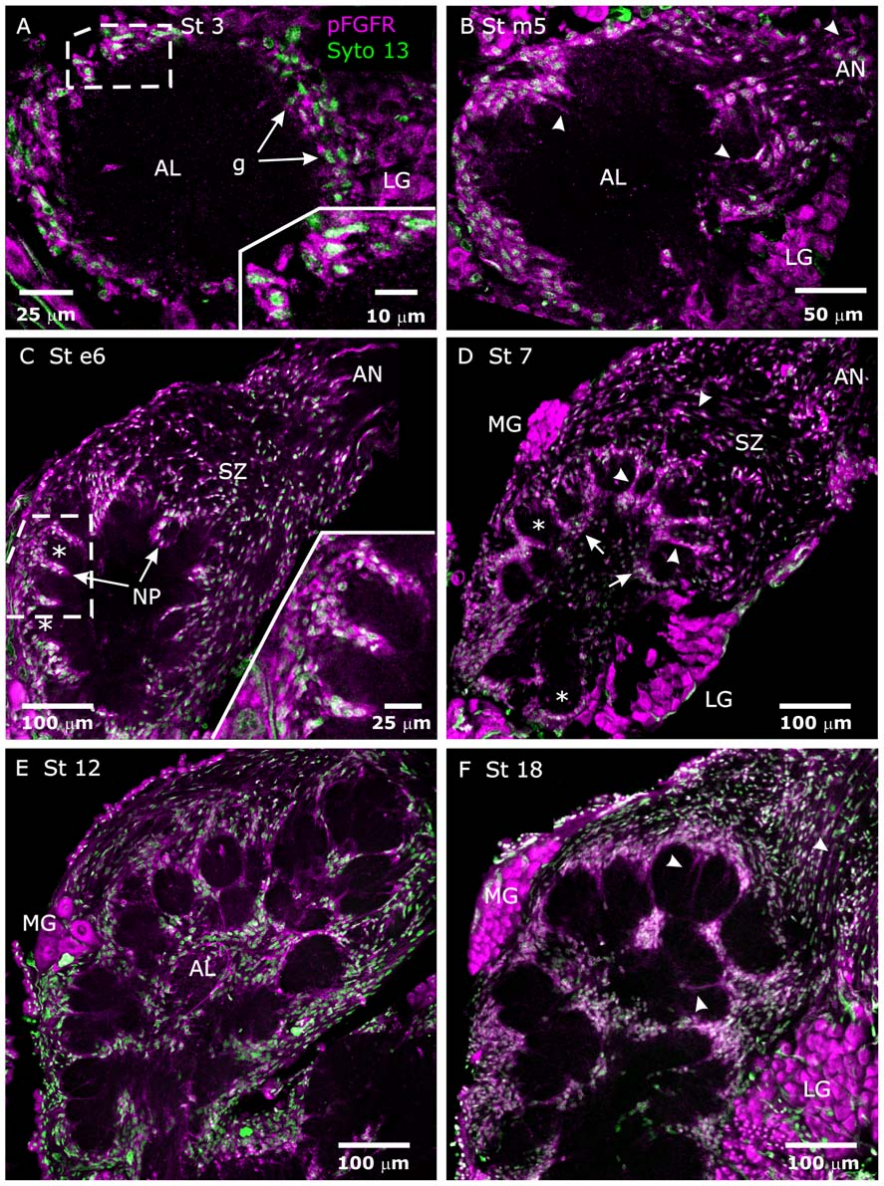
FGF receptors are expressed and activated on antennal lobe glial cells throughout adult development. **A–F:** Labeling with an antibody to activated (phosphorylated on Tyrosines 653/654) human FGFR1 (pFGFR, magenta) and with Syto 13 (green) to label cell nuclei showed that FGFRs are present and activated on the NP and SZ glia throughout adult development. By stage 7 (panel **D**), NP glial cell bodies (arrows) had migrated to surround developing glomeruli (*). Extended processes of SZ and NP glial cells were also clearly labeled (see **B,D,F**, arrowheads). LG, MG = lateral and medial group of AL neuron cell bodies. Projection depths = 2 µm in **A**, 3 µm in **B**, 5 µm in **C**, 10 µm in **D**, 12.5 µm in **E**, 15 µm in **F**.

**Figure 3:**
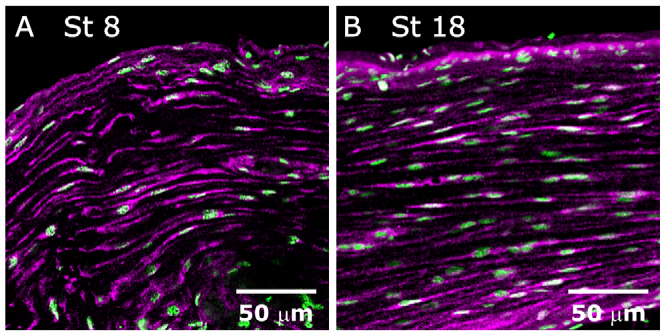
FGF receptors are also expressed and activated on antennal nerve glial cells throughout metamorphic adult development. **A,B:** pFGFR (magenta)/Syto 13 (green) labeling of antennal nerves at the middle (panel **A**) and the end (panel **B**) of adult development reveals activated FGFRs on AN glia both during (**A**) and after (**B**) the period of glial migration. Single optical sections (objective = 40× in **A**, 20× in **B**).

Labeling with an antibody to the activated (phosphorylated on tyrosines 653–654) FGFR reveals that that most, if not all, SZ, NP and antennal nerve (AN) glial cells express activated (phosphorylated) FGFRs throughout adult metamorphic development (Figs. 2,3). Cell bodies of most, if not all, AL neurons were labeled as well (Fig. 2, “MG” and “LG”), but we never detected labeling of their dendritic arbors in the antennal lobe neuropil. Panels D–F of Figure 2 clearly illustrates the large difference in size (both cell body and nucleus) between AL neurons of the medial and lateral groups (MG, LG) and all glial cells. This difference, plus the stereotyped locations of AL neurons (cell body clusters) and glial cells (sorting zone, surrounding glomeruli) allows for simple identification of glia and neurons [11], [16], [35].

**Figure 4:**
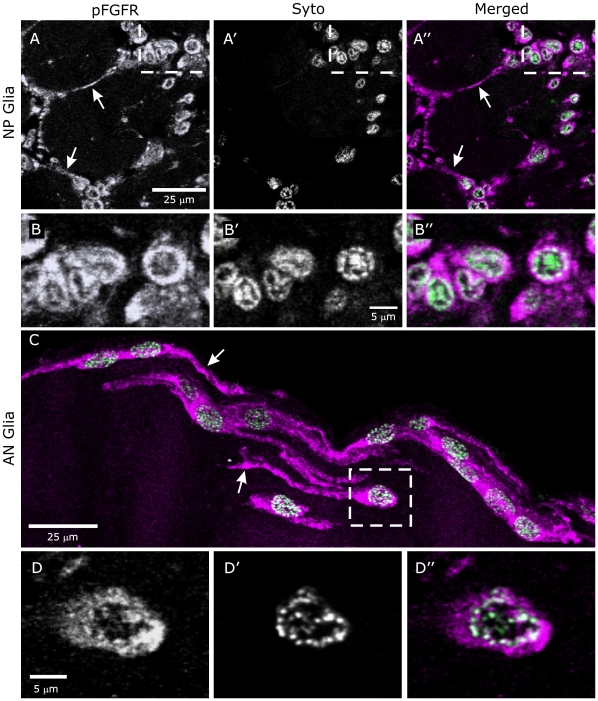
Activated FGFRs are present on glial cell membranes and in nuclei. **A,B:** Labeling for pFGFRs is present on NP glial processes (arrows) and associated with glial cell bodies (**A, A″**). Syto labels glial nuclei. **B–B″:** An enlarged view of the boxed area of **A″** demonstrates co-localization of labels for pFGFRs and DNA in glial cell nuclei. **C,D:** As for NP glia, AN glia label for pFGFRs both on their processes (arrows in **C**) and in their nuclei (**D″**). In both cases colocalization appears to be confined to DNA close to the nuclear envelope (**B″, D″**). Images are single optical sections (40× objective) except for **C** (16 µm stack).

As the projections shown in Figures 2 and 3 suggested a possible colocalization of pFGFR and cell nuclei, additional imaging was done at higher magnification. In single optical sections, both centrally derived (NP and SZ) and peripherally derived (AN) glia displayed labeling of processes, cell bodies, and nuclei (Fig. 4). It is interesting to note that the nuclear pFGFR labeling co-localized with Syto labeling of chromatin close to the nuclear membrane (Fig. 4B″,D″). Pre-adsorption of the antibody to phosphorylated FGFR with its blocking phospho-peptide essentially abolished labeling of glia and AL neuron cell-bodies (Fig. S2). Labeling with antibodies to the extracellular region of the FGFR and to HSPG, a necessary FGFR co-receptor [25], [29]–[30], mirrors the labeling for the pFGFRs in glial processes and nuclei and in AL neuron cell bodies and nuclei (Fig. S3). As was found for the pFGFR labeling (Fig. 2), these antibodies did not label AL neuron dendrites

**Figure 5:**
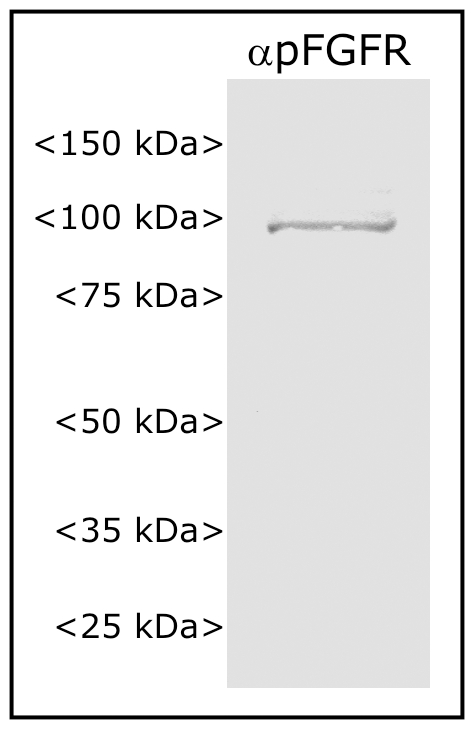
The antibody to the activated FGFR reveals a single band on western blots. Antennal lobe tissue, probed with the anti-pFGFR antibody, reveals a single band at ca. 98 kDa.

Two FGFRs (Heartless, with 2 Ig domains, and Breathless, with 5) are known in *Drosophila* [41]. In Lepidoptera, one FGFR sequence each is known for *Bombyx mori* and *Spodoptera frugiperda* [40]. Analysis of the amino acid sequences reveals that the Lepidopteran FGFRs, with calculated molecular weights of 97.3 and 96.1 kDa, respectively, possess 3 Ig domains (the pattern most often seen in vertebrate FGFRs, [24]). To characterize the FGFRs labeled in AL and AN glia of *M. sexta*, we performed western blots of antennal lobes plus antennal nerves. The anti-pFGFR antibody, when used on blots of tissue treated with a phosphatase-inhibitor cocktail, produced a strong band at 98 kDa (Fig. 5), in close agreement with predicted molecular weights of the two known Lepidopteran FGFRs. The slightly higher value is likely due to post-translational modification.

### Effects of Blocking FGFR Activation on Glial Cells

**Figure 6:**
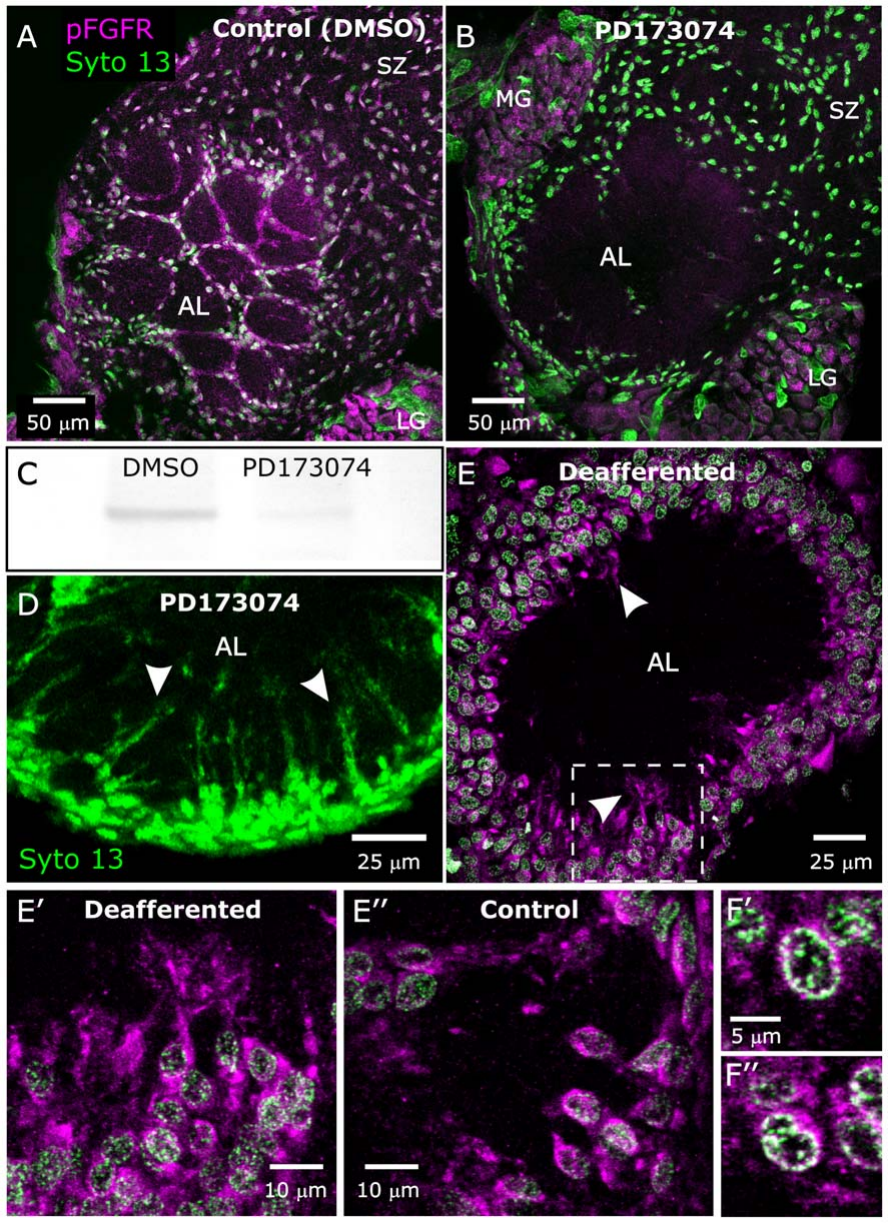
Blocking activation of the FGFR blocks migration of neuropil glia. **A,B:** Animals injected at stage 4 with DMSO or DMSO + PD173074 and examined at stage 7. **A:** Control animal injected with vehicle (DMSO) and labeled with the anti-pFGFR antibody (magenta) and Syto 13 (green) to show cell nuclei. Neuropil glial cells have migrated to surround glomeruli as in untreated animals. A more anterior view than those used in Fig. 2 was chosen to better illustrate the intense labeling of NP glial processes surrounding glomeruli. **B:** Animal injected with DMSO containing 0.5 mg PD173074. Labeling for activated FGFRs on glial cells is absent, and most neuropil-associated (NP) glia have failed to migrate to surround glomeruli. Sorting zone (SZ) glia have migrated normally despite their lack of labeling for activated FGFRs. **C:** A western blot of control and PD173074-treated antennal-lobe tissue from which neuronal cell bodies had been removed demonstrates a nearly complete absence of labeling for pFGFRs for the PD173074-treated lobes. **D:** Another animal treated with 0.5 mg PD173074 (beginning at stage 3), dissected at stage 7, and labeled with Syto 13. At increased gain, some NP glial processes can be seen to have extended into the neuropil (arrowheads) despite the absence of cell-body migration. **E:** A stage-6 antennal lobe from an animal chronically deprived of ORN innervation on one side (antennal anlagen removed at stage 1.) Although lack of ORN innervation resulted in lack of glial migration, glial cells did exhibit activated FGFRs (magenta, arrowheads). **E′:** Enlarged section from boxed area of panel **E** better illustrates the labeling of glial processes. **E″:** Opposite lobe, which was not deprived of ORN input, appears to have the same intensity of pFGFR labeling. **F′,F″:** Individual cells of deafferented (**F′**) and control (**F″**) lobes both display colocalization of pFGFR and DNA labels. LG, MG = lateral and medial group of AL neuron cell bodies. Projection depths = 10 µm in **A, B, D, E, E′**. **F′, F″** are single optical sections (40× objective).

Sorting zone glial cells respond to the arrival of ORN growth cones by proliferating and migrating to form the sorting zone, and NP glial cells respond to formation of protoglomeruli by ORN axons by extending processes and migrating to form a glial envelope around each protoglomerulus. We asked if glial FGFRs might play a role in these events, which take place predominantly between stages 3 and 8 of adult development. We used the FGFR-specific tyrosine kinase inhibitor, PD173074 [36], [37], to block activation of glial FGFRs. PD173074-treated animals showed a loss of activated FGFRs on glial cells of the primary olfactory pathway, as measured by pFGFR immunocytochemistry at stage 7 (Fig. 6A,B) and by western blot using stage-e6 antennal lobe tissue from which the neuronal cell body clusters had been removed (Fig. 6C). Interestingly, although all three classes of glia examined here (AN, SZ and NP) normally exhibit activated FGFRs (Figs. 2, 3, 4), only the NP glia (which normally migrate to surround glomeruli) displayed a lack of migration following blockade of FGFR activation (Fig. 6B). In contrast, the population of SZ and AN glia appeared to have migrated normally following PD173074 treatment. Imaging of nucleic acid labeling of NP glia at higher photomultiplier gain revealed that the NP glial cells had, in fact, extended processes into the antennal lobe neuropil (Fig. 6D). This suggests that FGFR activation in NP glia may be necessary to couple glial cell-body motility to the arrival of ORN axons or to couple glial cell-body motility to process extension, but not to initiate process extension. Because migration of NP and SZ glia normally requires the presence of ORN axons [11], [42], we checked for activation of FGFRs in animals deprived of ORN innervation, in which NP glial cells fail to migrate. Labeling for the activated FGFR was present in glial cells in both the innervated and chronically non-innervated antennal lobes (Fig. 6E–F”).

**Figure 7:**
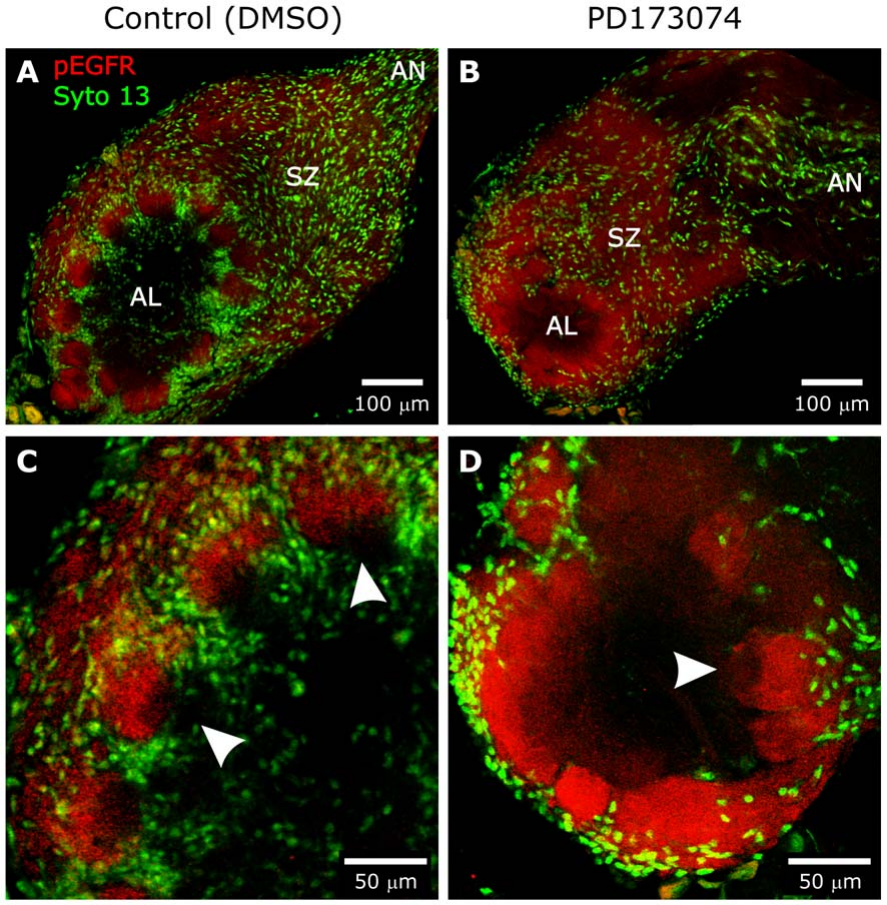
A,B: Animals treated as in Figure 6A,B and examined at stage 8. Labeling with an antibody to activated Epidermal GFRs (red) reveals a normal pattern of activation in the ORN axons demonstrating that PD173074 did not affect the Epidermal GFRs. **C,D:** Enlarged images from the same animals demonstrate that the axons occupy their normal territory within the apical half of glomeruli in the control AL (arrowheads in panel **7C**) and in the outer portion of the neuropil in the PD173074-treated AL (arrowhead in panel **7D**), where glomeruli are demarcated only by glial processes, not by glial cell bodies as in normal lobes. Depth of projections was 10 µm.

PD173074 has been shown to be a specific inhibitor of FGF receptors in vertebrates [36], [37]. A similar specificity has not been demonstrated in invertebrates, so we wanted to make sure that the drug was not acting via other receptor tyrosine kinases. We previously have shown EGFR activation to occur transiently on ORN axons as they traverse the sorting zone and form glomeruli [18]. When control and PD173074-treated animals were labeled with an antibody to the activated epidermal growth factor receptor, ***EGFR*** (Fig. 7), they displayed the normal pattern of labeled ORN axons in the sorting zone and glomeruli [18], suggesting that PD173074 does not interfere with EGFR activation in *M. sexta*.

### Effects of Blocking Glial FGFR Activation on AL Neurons

**Figure 8:**
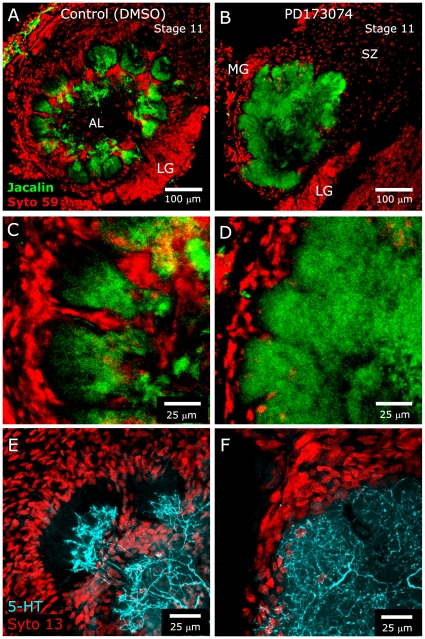
Blocking glial FGFR activation leads to over-extension of AL neuron dendrites. Animals were injected with DMSO or DMSO containing 0.5 mg PD173074 beginning at stage 3. **A,C:** Control animals examined at stage 11 display characteristic Jacalin labeling (green) of AL neuron dendrites in the basal region of glomeruli. Glial nuclei and AL neuron cell bodies are labeled with Syto 59 (red). **B,D:** Treated animals examined at stage 11 exhibit a lobular, rather than glomerular, arborization. AL neuron dendrites extend beyond their normal, mostly basal territory in the glomeruli, reaching almost to the glial border (compare **C,D**). **E,F:** Animals treated as in **A–D** and labeled for 5-HT (blue) and Syto 13 at stage 12 reveal differences in dendritic arborization of the sole serotonergic neuron similar to those seen in panels **A–D** (i.e. dendrites overextending in the PD173074-treated animals). Projection depths = 25 µm in **A–D**, 26 µm in **E,F**.

Because glial cell–neuron communication is often reciprocal [9], we wanted to know if blocking FGF receptor activation on glial cells might affect glia-to-neuron communication within the antennal lobe, resulting in alteration of neuronal growth patterns. In normally developing animals, NP glia stabilize the protoglomeruli, which then allows the subsequently ingrowing AL neuron dendrites to develop their characteristically tufted arbors and allows the neuropil to become glomerular in organization [12]. We also have shown previously that glial processes alone, in the absence of glial cell body migration, are sufficient to establish boundaries within the neuropil ([15]). In the current study, we examined general AL neuron growth patterns by labeling dendrites with the lectin, Jacalin [43], as well as the arborization of the sole serotonergic (5-HT) AL neuron, which extends dendrites into all glomeruli. In control (DMSO-treated) animals examined at stage 11–12, after the completion of axon ingrowth at stage 9, Jacalin and 5-HT labeling was observed predominantly in the basal (inner) portion of glomeruli, where AL neuron dendrites are concentrated (Fig. 8A,C,E). In PD173074-treated animals examined at the same stages, the neuropil clearly was not glomerular, but it did have a lobular organization (Fig. 8B,D,F), presumably maintained by glial processes that did extend (Fig. 6C), despite the lack of glial cell body migration. Significantly, AL neuron dendrites were not confined to the basal portion of the lobules but extended outward to the glial borders (Fig. 8D,F). This result suggests that activation of FGFRs on NP glia leads to signaling that is important in defining the extent of AL neuron arborization in the glomeruli.

### Effects of Blocking Glial FGFR Activation on Glial Survival and Proliferation

**Figure 9:**
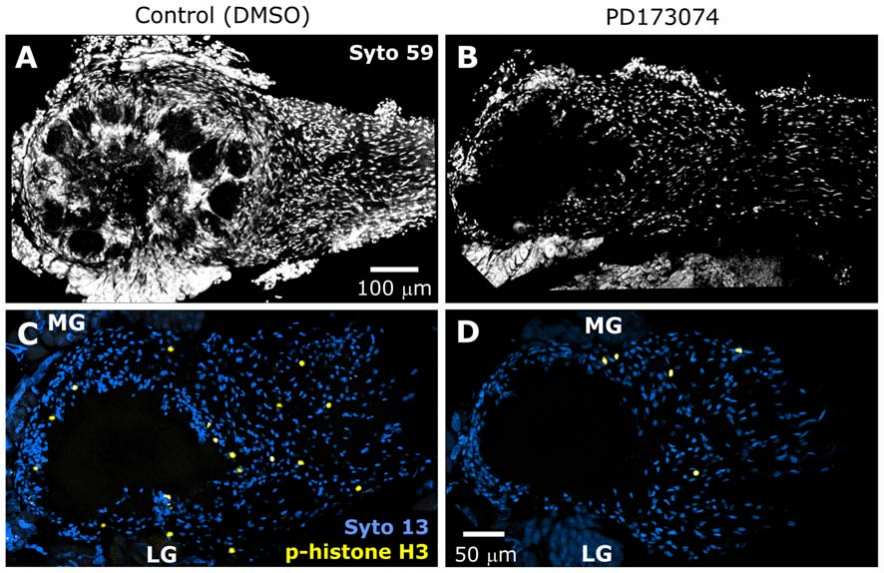
PD173074 treatment results in reduced glial numbers at later stages. **A,B:** Syto 59 labeling of the same antennal lobes shown in Figure 8A–D. At lower magnification, Syto 59 labeling of cell nuclei illustrates the significant reduction in cell number in treated animals at later stages (stage 11 shown). The reduction occurs in regions normally occupied solely by glial cells. **Blocking glial FGFR activation leads to a decrease in proliferation.** **C,D:** Control and PD173074-treated animals were allowed to develop to late stage 5, then dissected and their brains labeled with an antibody to phospho-histone H3, an indicator of mitosis (yellow). Syto 13 (blue) was used to visualize all glial nuclei. Projection depths = 30 µm in **A,B** and 10 µm in **C,D**.

Comparison of the above preparations at lower magnification reveals a visually obvious difference in glial cell number by stage 11 (Fig. 9A,B) following blockade of FGFR activation, whereas this decrease in cell number was not apparent at earlier stages (Fig. 6). Because FGFR activation is known to be important in cell proliferation and survival [24], [25], and because the presence of sufficient SZ and NP glia is essential for ORN axon sorting, fasciculation, and targeting, and for glomerulus stabilization during development [12], [14], [16], it was important to understand the time course of the reduction in glial number. We examined both glial proliferation via labeling for phosphorylation of histone H3 (indicative of cell division, [44]) and glial death via TUNEL labeling (an apoptosis marker) in control and treated animals at several stages of adult development. As mentioned earlier, cell types in the primary olfactory pathway of *Manduca sexta* have been well characterized as to position and size. In discussing our results concerning cell division and apoptosis, we therefore refer to cells as glia or neurons based on position and size, but cannot rule out the unlikely possibility that treatment with PD173074 may have resulted in the presence of cells not normally found in the primary olfactory pathway or in migration of cells to atypical locations.

#### Phospho-histone H3

We labeled for phospho-histone H3 (phH3), which marks cells undergoing mitosis at the time the brain was removed and fixed. This is expected to produce a smaller number of labeled cells than was found in our previous studies of NP glial cell division using either BrdU [16], [45] or ^3^[H]-thymidine [46], which labeled the cells that were undergoing DNA replication over a 6–18 hr period. Animals were injected once or twice (24 hr apart) with DMSO alone or PD173074 (0.5 mg in DMSO), and were allowed to develop to stage mid-5 (1× injected) or late-5 (2× injected), then processed. Typically, the number of phospho-histone H3-positive glial nuclei in the olfactory pathway was small, ranging from 4 to 40 in a given 100-µm vibratome section and from 0 to 4 in a single optical section (Fig. 9C,D), but the total number of glial cell nuclei in a 100-µm-thick section was sufficiently large that we chose to count cells in single optical sections. For each animal, we imaged through the central region of the olfactory pathway, counting numbers of positive nuclei per optical section (2.5 µm z-step), taking care that the same glial nuclei were not counted twice. We calculated the average number of phH3-positive glial nuclei per optical section as well as the average total number of glial cells (SZ, NP and nerve layer) in 2–3 non-adjacent optical sections. From the average values obtained, we calculated the ratio of phH3-positive nuclei to total number of nuclei. The ratios were then compared between control and PD173074-treated animals. We found that proliferation continued in both control and treated animals, but the PD173074-treated animals showed fewer dividing cells compared to control for both the 1× and 2× injection protocols, respectively (Table 2). All dividing cells were in locations normally occupied solely by glial cells. Antennal-lobe neurons are known to have undergone terminal differentiation prior to the events initiating formation of the antennal lobes [14], and thus were not expected to, and did not, label with the phH3 antibody.

**Table 2:** Effect of PD173074 on SZ+NL+NP glial cell division.

Treatment	Vibratome sections examined	Optical sections examined	Optical sections quantitated	Avg glia/optical section	Avg phH3^+^ nuclei/op sec	phH3^+^ nucleiper 1000 glia (StdDev)	Reduction in glial division with PD173074
1XDMSO (n = 2)	6	107	13	210.3	0.95	4.6 (1.8)	
1XPD (n = 3)	6	108	12	167.5	0.27	2.0 (1.4)	57% (p<.0005)
2XDMSO (n = 2)	8	128	16	358.0	1.85	5.3 (1.2)	
2XPD (n = 3)	11	170	21	183.3	0.4	2.1 (1.2)	60% (p<.0001)
Totals	31	513	62				

Total glial nuclei counted: 14,428.

p-values obtained using Student's *t*-test.

#### Apoptosis (TUNEL labeling)

**Figure 10:**
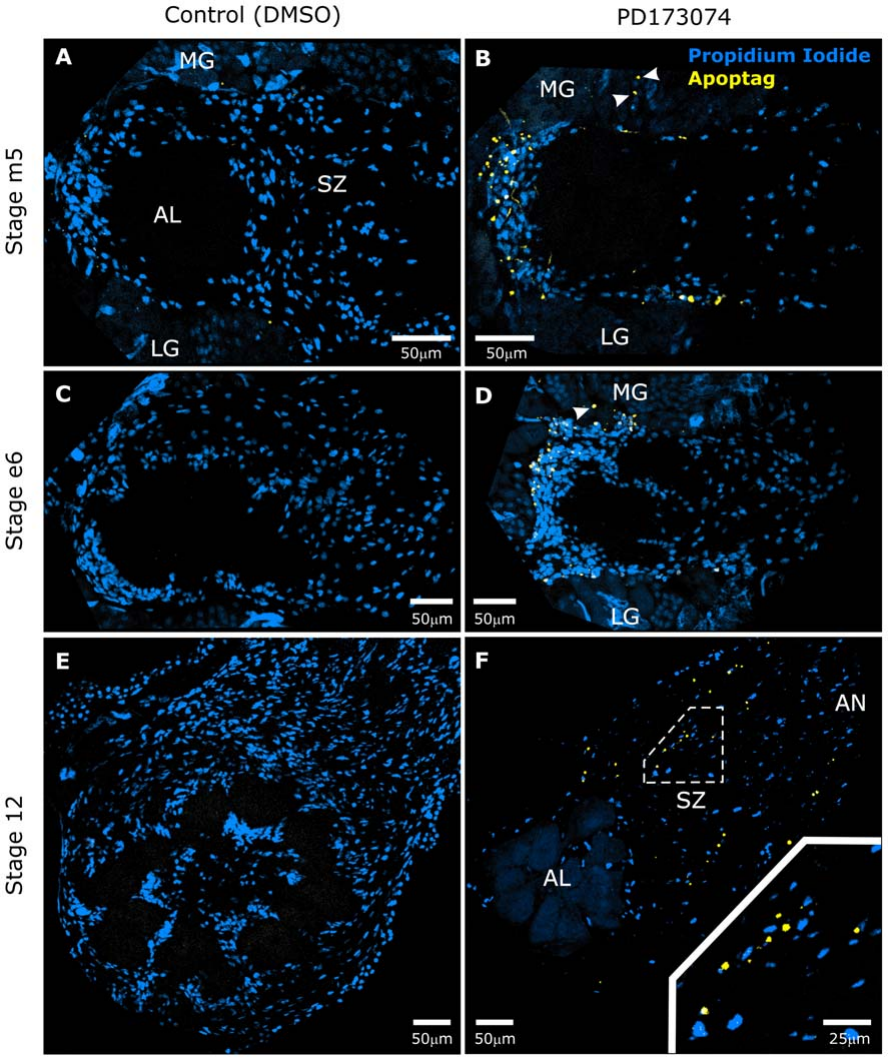
Blocking glial FGFR activation leads to apoptosis. **A–F:** Control and PD173074-treated animals were allowed to develop to various stages, then dissected and analyzed for apoptotic nuclei using the TUNEL technique. Numerous apoptotic nuclei (yellow) were seen in treated animals (panels **B,D,F**), but few to no apoptotic nuclei were seen in control animals (panels **A,C,E**) at all stages examined. Propidium iodide (blue) was used to visualize all cell nuclei. Arrowheads in panels **B,D** indicate apoptotic nuclei among the medial group of antennal lobe neurons. Inset in panel **F** shows a higher magnification view of a region within the sorting zone of the antennal nerve. Projection depths = 10 µm.

To determine the contribution of apoptosis to the reduction in cell number, TUNEL assays were performed on control and treated animals at various stages of development. At stages mid-5 and early-6, we found evidence of considerable apoptosis of NP (but not SZ or AN) glial cells in treated but not in control animals (Figs. 10A–D, S4, Table 3). A small number of apoptotic nuclei also were seen among the medial group of AL neurons (arrowheads in Fig. 10B,D) in PD174074-treated animals. Because clusters of neuronal cell bodies also include cortical glial cells [35], apoptotic nuclei found in the cell body clusters could be either neuronal or glial. In either case, the number of apoptotic nuclei in these locations was very small and the overall size of the clusters was not obviously diminished suggesting that, if present, neuronal apoptosis was minimal. Also, a previous study [47] showed that removal of the medial group projection neurons from stage 4 forward had no effect on the glomerular organization of the antennal lobe.

**Table 3:** Effect of PD173074 on Apoptosis.

Number of Apoptag-positive AL+AN nuclei per frozen section.			
Stage	mid-5	early-6	12
Control	n = 5	n = 7	n = 6
Median	2	0	0
Avg (S.D.)	1.2 (1.1)	0.3 (0.5)	0 (0)
PD173074	n = 18	n = 16	n = 12
Median	16.5	40	12.5
Avg (S.D.)	18 (6.4)	38.9 (14.1)	14.7 (8.9)

Note: Cells undergoing apoptosis were limited to regions normally occupied solely by NP glia at stages mid-5 and early-6. At stage 12, apoptotic nuclei were found in the sorting zone and antennal nerve (see Fig. 10). “n” = number of frozen sections examined.

Interestingly, we also found no evidence for apoptosis among SZ glial cells at stages 5–6, even though, like the NP glia, SZ glial cells are centrally–derived. Treated animals allowed to develop to stage 12 displayed the sparse population of NP glia seen previously (Fig. 9B), and many apoptotic nuclei now appeared in regions normally occupied solely by SZ and AN glial cells (Fig. 10F). Control animals at all stages displayed a normal glial population with few or no apoptotic nuclei (Fig. 10A,C,E, Table 3).

Considered together, the results described above indicate that blocking of FGFR activation leads both to glial cell apoptosis and to reduction of glial cell proliferation. The effect on cell number is minimal at early stages (5–6), but becomes significant at later stages.

### Effects of Blocking Glial FGFR Activation on ORN Axons

We next examined ORN axon growth patterns following blockade of glial FGFR activation. The effects described below were not due to a non-specific effect of PD173074 on the activation of the axonal **E**GFR, as shown earlier (Fig. 7).

**Figure 11:**
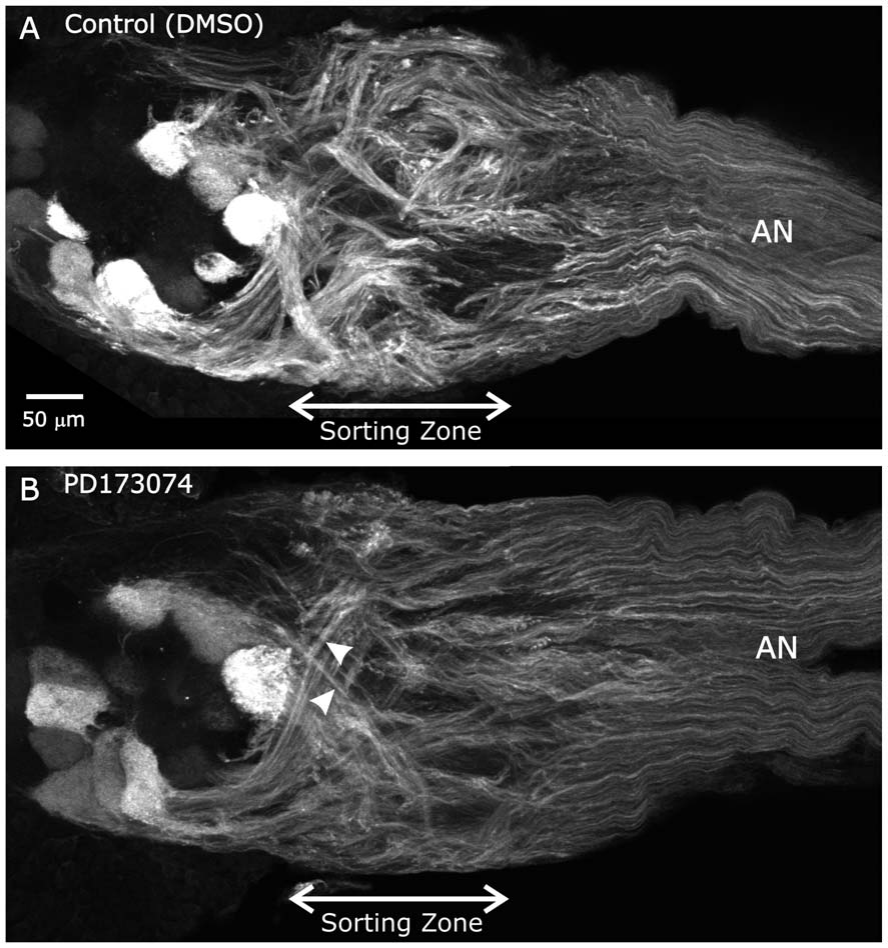
Blocking glial FGFR activation leads to abnormal fasciculation of ORN axons in the sorting zone. Control and PD173074-treated animals were allowed to develop to stage 6–7, then dissected and their brains labeled with an antibody to *M. sexta* Fasciclin II in order to visualize the distribution of a known subset of ORN axons. **A:** In control animals, ORN axons normally exhibit significant changes in fasciculation (relative to their state in the antennal nerve) a short distance into the sorting zone. **B:** PD173074-treated animals exhibited unchanged fasciculation in traveling through the sorting zone, although they did show increased fasciculation on exiting the sorting zone (arrowheads). Projection depths = 35 µm in **A**, 45 µm in **B**.

We visualized a subset of ORN axons that are MFasII-positive to examine the trajectories and fasciculation of these axons in the sorting zone and their distribution in the antennal lobe. These axons are known to target 14–21 of the 62–64 glomeruli in the antennal lobe [17]. Normally, MFasII immunocytochemistry at stage 6 reveals a dramatic change in axon trajectory and fasciculation a short (50–70 µm) distance into the sorting zone, clearly illustrated in the untreated antennal lobe shown in Figure 1B. These changes are glia-dependent [16]. In animals treated with DMSO or PD173074 and examined at stage 6–7, compact and sharply-defined axon fascicles formed a short distance into the sorting zone in the DMSO-treated animals (Fig. 11A), but appear not to have formed until the axons were emerging from the sorting zone in PD173074-treated animals (arrowheads in Fig. 11B). Fasciclin labeling in the antennal nerve distal to the sorting zone appears similar in both panels, suggesting that differences in labeling in the sorting zone are truly due to lack of fasciculation in PD173074-treated animals rather than to downregulation of Fas expression. Counts of SZ glial cells in single optical sections of these stage-6–7 preparations revealed no difference in glial number between control and treated animals (300±30). The lack of a difference in SZ glial number in this experiment is consistent with the absence of apoptosis among SZ glial cells at early stages (Fig. 10) and a low level of proliferation in the total (NP, SZ, and NL) glial population (Table 2). The diameters of the antennal nerves of control and PD173074-treated animals in the sorting zone region are similar, as shown in Figure 11A,B, suggesting that the number of ORN axons reaching the brain was affected minimally, if at all, by PD173074.

Because we saw no decrease in SZ glial number through stage 6, by which time the events important to ORN axon fasciculation have occurred, the results above suggest that differences in ORN axon growth patterns are attributable to reduced or altered signaling from a normal complement of glia rather than to reduced signaling due to a smaller complement of glial cells, the latter decreasing the possibility for neuron-glial cell interaction.

Because of the dramatic effect of PD173074 treatment on ORN axon fasciculation in the sorting zone, it was important to ensure that the effect was due to blocking glial FGFR activation and not to blocking FGFRs present on ORN axons. We looked closely at ORN cell bodies in the antenna. Using imaging parameters optimized for pFGFRs in AN glia (Fig. S5A), we scanned longitudinal- and cross-sections of antennae. ORN cell bodies were negative for pFGFRs (Fig. S5B,B′). Similarly, the antennal nerve distal to the sorting zone exhibited no pFGFR labeling of the ORN axons (Fig. S5C,C′). Thus the immunocytochemical evidence argues against expression of FGFRs by ORNs and suggests that effects of PD173074 treatment on ORNs is mediated indirectly via effects on glia.

### Effects of Blocking Glial FGFR Activation on Targeting and Termination of ORN Axons

**Figure 12:**
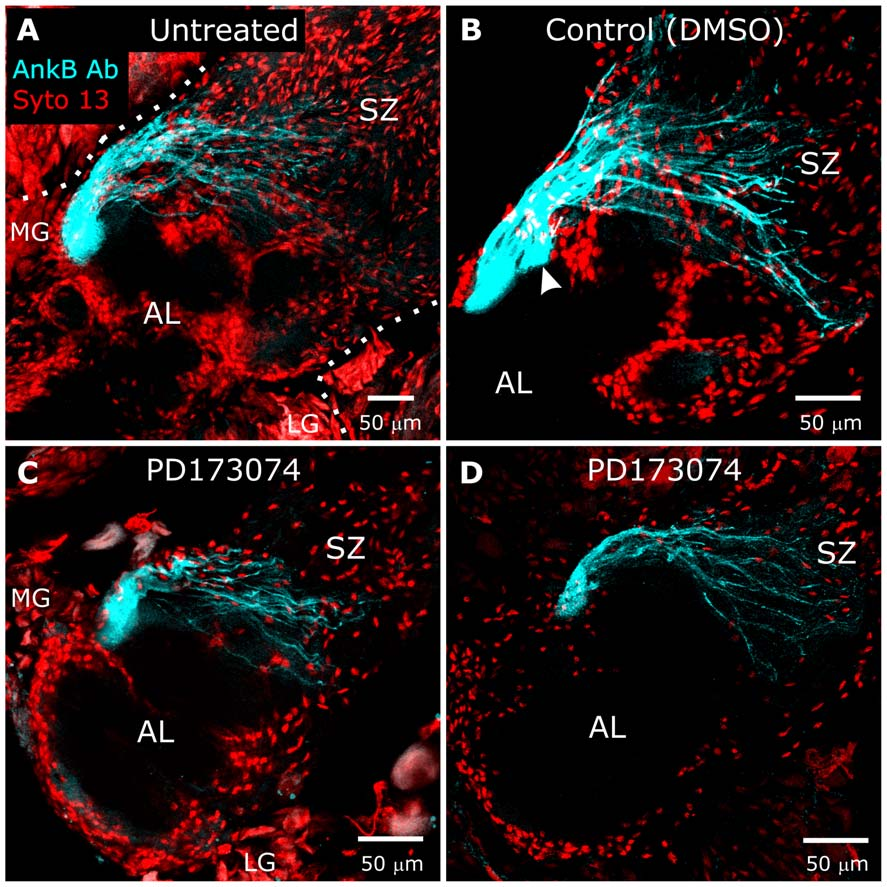
Blocking glial FGFR activation does not greatly perturb targeting of ORN axons to a specified glomerulus. Brains from untreated animals (**A**), control (DMSO, **B**), and animals injected with PD173074 in DMSO beginning at stage 3 (**C,D**) were labeled at stage 7 with Syto 13 (red) to identify glial nuclei and AL neuron cell bodies. Axons targeting a single glomerulus were labeled with an antibody produced against human Ankyrin B (blue), though the *Manduca* protein recognized by this antibody is unknown [33]. Although treated animals displayed a lack of NP glial cell body migration in the antennal lobe, axons labeled with the Ankyrin B antibody were seen to project to a single region (**C,D**) adjacent to the medial group of AL neurons, as they do in untreated and control animals (**A,B**). Dotted lines in **A** outline the antennal lobe and AL neuron cell groups (LG & MG). Arrowhead in **B** points to a large fascicle which merged with the main fascicle to form a single glomerulus in the adjacent section. Projection depths = 30 µm in **A**, 22.5 µm in **B**, 12.5 µm in **C**, 20 µm in **D**.

During embryonic development, glial cells have been shown to play major roles as guidepost cells, causing abrupt changes in axon trajectories via molecules such as slit, netrin, and commissureless [1]–[10], [48], [49]. To determine whether the glial FGFR might be involved in the process by which ORN axons correctly sort together and target a given glomerulus, we blocked FGFR activation and used the subset of axons targeting a uniquely identifiable glomerulus as an assay. The axons targeting this glomerulus, referred to as “glomerulus X,” label with an antibody to human Ankyrin B [33]. The stereotypical location of glomerulus X adjacent to the primary neurite tract of the medial group of AL neurons allows us to ask if a particular intervention can perturb the convergence of anti-ankyrin-immunoreactive ORN axons to a glomerulus in this location. The labeled ORN axons in untreated (n = 11), vehicle control (n = 3), and PD173074-treated (n = 7) animals always targeted a single location (Fig. 12). As expected (Figs. 6, 7, 8, 9, 10), the treated antennal lobes showed minimal NP glial cell body migration along glomerular boundaries, but the labeled axon terminal branches always clustered adjacent to the primary neurite tract of the medial group of AL neurons, as they did in controls. It therefore appears that, at least for this subset of axons, molecules needed for their correct targeting are produced independently of glial FGFR activation, or are produced at a time prior to stage 3, when the animals were injected with PD173074.

The over-extended arborization of dendrites of AL neurons in PD173074-treated animals seen in Figure 8B,D,F raised the possibility that the dendrites were extending into glomerular territory normally occupied mainly by terminal branches of ORN axons. However, labeling (pEGFR antibody) of ORN axon terminal fields in the antennal lobes of control and PD173074-treated animals at stage 7–8 (Fig. 7C,D) reveals that ORNs in treated animals formed and maintained terminal arborizations in the normal apical portion of each glomerulus, constrained by the glial processes that form an envelope despite the lack of NP glial cell body migration (Figs. 6D, 8). We know from previous experiments that without those processes, the axon terminal branches would spread laterally into the territory of adjacent axons and glomerular organization would be lost [12]. Thus the overextension of AL neuron dendrites into the apical portion of the developing glomeruli is unlikely to be due to an abnormality in the distribution of the ORN axon terminal branches.

## Discussion

The essential nature of FGFR-mediated signaling for cell differentiation, proliferation, survival, migration, and shape has been well documented in vertebrates and invertebrates [24], [25], [37], [50]–[54]. In insects, primary cultured *Drosophila* embryonic neurons display Neuroglian- and Fasciclin II-dependent neurite outgrowth mediated via Heartless [39]. In intact *Drosophila* embryos, Heartless has been found to be necessary for directional (but not random) migration of mesodermal cells [50]–[52]. In the developing adult ocellar sensory system, Heartless works with the EGF receptor in Neuroglian-mediated OP and BM axon extension and guidance, in which the EGFR appears to determine axon extension and Heartless dictates direction [22]. For glial cells in *Drosophila* embryos, Heartless has been shown to be necessary for migration of longitudinal glia and for their ability to enwrap longitudinal axon tracts [54]. Similarly, in development of the adult *Drosophila* visual system, Heartless expressed by CNS glia is activated by glial-cell-derived Pyramus and photoreceptor-axon-produced Thisbe to cause proliferation and migration outward along the optic stalk followed by glial differentiation and wrapping of axons in the optic disc [55], [56].

We find that in the primary olfactory pathway of *M. sexta*, glial cells of all types express FGFRs, and that these FGFRs are activated throughout metamorphic adult development (Fig. 2). The question addressed here is whether FGFR signaling underlies some of the critical neuron-glial interactions that we have demonstrated at the cellular level to be required for normal development of the olfactory pathway. We took advantage of the fact that, as is the case for its human homolog, activation of the *M. sexta* FGFR can be blocked by the specific inhibitor, PD173074, as shown by the loss of labeling of glia and antennal-lobe western blots using an antibody that recognizes only the activated form of the receptor (Fig. 6B,C).

Western blot analysis suggests, based on size, that the *M. sexta* FGFR has three Ig domains, as is the case for two other known Lepidopteran FGFRs [40] and many vertebrate FGFRs [24]. The fact that an antibody to a highly conserved region of the FGFR tyrosine kinase domain produces a single band on western blots (Fig. 5), in conjunction with the fact that only one FGFR has been found in *Bombyx* and *Spodoptera* [40], suggests that Lepidopterans express only one FGFR. This is in contrast to *Drosophila*, which expresses two FGFRs: Heartless, with 2 Ig domains, important in development and organization of mesodermal structures including heart and somatic muscles in the embryo, and Breathless, with 5 Ig domains, important in development of the tracheal system of the embryo [41], [50]–[52], [54], [57]. Heartless is expressed in longitudinal glial cells [54] and both FGFRs are important in embryonic CNS development. The only other evidence for involvement in the post-embryonic CNS was reported in a brief study of 3^rd^ instar *Drosophila* in which Heartless, but not Breathless, mRNA was found in eye-antenna imaginal discs [58]. The current work in *Manduca* focuses on the developing adult, rather than embryonic or larval stages, however, making comparison with the *Drosophila* studies difficult. The important point here is that, in metamorphic adult development in *Manduca*, the FGFR is expressed by CNS and peripheral glia, and not by tracheae. High magnification imaging of antennal-lobe and antennal-nerve glia revealed the presence of FGFRs on glial processes but also closely associated with nuclear DNA (Fig. 4). DNA labeled with Syto 13 appears to be concentrated into “chromosome territories” [59] associated with intranuclear pFGFRs. We are not aware of other descriptions of nuclear localization of FGFRs in invertebrates, but this phenomenon has been described in cultured fibroblasts [60] and in human astrocytes and glioma cells, where nuclear localization appears to be correlated with transcriptional regulation and subsequent glial-cell proliferation [61], [62]. Further work is needed to determine whether or not nuclear localization of FGFRs can be connected to specific cellular functions in invertebrates.

Heartless expression also has been reported in embryonic *Drosophila* neurons grown in culture and *in vivo* [39]. We likewise saw evidence of FGFRs in the AL neurons, but only in their cell bodies, not in their dendrites (Fig. 2) or axons (not shown). There is evidence that FGFRs can be imported directly from endoplasmic reticulum to the nucleus without ever being expressed on the plasma membrane [62]. This latter phenomenon, termed “integrative nuclear FGFR signaling” may be relevant to our observation that FGFR labeling in the AL neurons is limited to their cell bodies, and might help explain why AL neuron cell bodies in PD173074-treated animals continue to label for activated FGFRs (Fig. 6B). In this scenario, activation of signaling pathways within AL neurons would lead to direct translocation of FGFRs from the endoplasmic reticulum to the nucleus in order to modulate gene transcription. The nature of the role of FGFRs in AL neurons remains unanswered.

Heparan sulfate proteoglycans have been described as essential co-receptors for FGFs [25], [29]–[30]. As was the case for pFGFRs, we found HSPGs expressed in glial cells and AL neurons (Fig. S3). Additionally, we found HSPGs both on cell processes and in nuclei. This, too, is in agreement with published accounts that HSPG localization can vary [63], [64].

We have shown previously that ORNs express ***E***GFRs [18] and find here that these EGFRs are activated normally following treatment with PD173074 (Fig. 7). If ORN EGFR activation had been blocked, ORN axons would have stalled in the sorting zone, making it thicker than normal [18]. The fact that antennal lobes of control and treated animals display sorting zones of comparable diameter indicates that ORN axons did not stall in the sorting zone, as they do when EGFR activation is blocked with PD168393 [18]. This supports the conclusion that PD173074 does not block EGFR activation in *M. sexta*.

We lack an antibody for the activated form of the only other *Manduca* receptor tyrosine kinase characterized to date, the Eph receptor [65], so we could not check for its possible inactivation. However, PD173074 was designed to competitively bind to the ATP-binding pocket of the FGF receptor, and amino acid alignments show that the Eph receptor lacks 8 of the 18 amino acids at specific locations needed to form the binding pocket for PD173074 (Fig. S6). Thus PD173074 appears an unlikely candidate for binding to and blocking activation of the Eph receptor.

Because it was important to determine whether the altered fasciculation of ORNs traversing the sorting zone in PD173074-treated animals was a ***direct*** result of blocking ORN FGFR activation, we also looked for evidence of expression of FGFRs by olfactory receptor neurons (Fig. S5). We found no evidence for pFGFRs in ORN cell bodies, axons, or dendrites within antennal sensilla, suggesting that the altered behavior of ORN axons in the sorting zone is the consequence of interrupting an interaction between the ORNs and glial cells that depends on FGFR activation in the glial cells.

### Blocking glial FGFR activation: effects on glia

#### Migration

During development of the olfactory pathway, glial migration occurs in response to the arrival of ORN axons and leads to the formation of the sorting zone and formation of the glial envelopes that stabilize developing glomeruli [4], [5], [9]. We have observed previously that NP glia fail to migrate but do extend processes following blockade of neuron-to-glial cell signaling via nitric oxide [15] or disruption of sterol-rich membrane subdomains with methyl-β-cyclodextrin [33]. We have shown here the same phenotype in PD173074-treated animals (Fig. 6D). Together, these several observations indicate that glial cell migration in response to ORN axon ingrowth and coupling of cell-body movement to process extension depends on several signaling systems, including FGFR activation.

As background for assessing the connection between FGFR activation and NP glial cell migration, we know the following: 1) NP glial cells migrate only if a sufficient number of ORN axons have arrived at the antennal lobe [11], [42]. 2) NP glial migration depends on influx of extracellular calcium through voltage-gated calcium channels following depolarization [66]. 3) These calcium channels are activated by the presence of ORN axons; they are not activated until *after* initial contact with ORN axons (stage 5 for SZ glia, stage 6 for NP glia) and glia in antennal lobes deprived of ORN innervation do not exhibit functional voltage-gated calcium channels [66]. 4) NP and SZ glia express nicotinic acetylcholine receptors; blocking these receptors *in situ* eliminates calcium transients in response to carbamylcholine, an acetylcholine receptor agonist [67]. Thus both NP and SZ glia are capable of responding to ORN axon-derived acetylcholine via depolarization and activation of the voltage-gated calcium channels, an essential prerequisite for migration. 5) NP glia imaged *in situ* display no calcium influx in response to 200 µM carbamylcholine at stage m5, show maximum influx at stage 6, at the height of glial migration, and then display an influx that declines to about half maximum by stage 9, indicating a strong temporal correlation between acetylcholine-induced glial calcium influx and glial cell migration to surround protoglomeruli [67]. In the context of our results that FGFR activation is coupled to NP glial cell migration, the above observations raise the intriguing possibility for future study that glial FGFR activation, modulated by arrival of ORN axons, leads to expression or functionality of voltage-gated calcium channels and/or nicotinic acetylcholine receptors on NP glial cells. Alternatively, pathways downstream of calcium influx and FGFR activation could intersect to produce glial cell migration via, for example, activation of doublecortin, src-family kinases, and focal adhesion kinases [68]–[71].

In contrast to the effect on NP glial cells, pharmacologic blockade of FGFR activation did not prevent the migration of SZ or AN glial cells (Figs. 6B, 7B). Blockade of ORN-mediated nitric oxide signaling [15] or disruption of sterol-rich membrane subdomains with methyl-β-cyclodextrin [33] also failed to block SZ glial cell migration. Our inability to block SZ glial migration by these various methods may be due to the fact that the initial contact between ORN growth cones and the glial cells that become SZ glia occurs late in stage 3, and thus the signaling necessary for SZ glial migration may have occurred before the various drug treatments could take effect. Injecting drugs at earlier stages generally results in developmental arrest (Gibson, unpublished). Another possibility is that redundancy in the signaling pathways that elicit SZ glial cell migration ensures formation of this critical region in the olfactory pathway. As for the continued migration of AN glia in PD173074-treated animals in the *Manduca* system, similar results have been reported in *Drosophila* antennal nerves in which glial cells express a dominant-negative form of Heartless [72]. We have found AN glia to express EGFRs as well as FGFRs [18]; it is possible that they depend on EGFR activation for migration and FGFR activation for survival.

#### Survival

Activation of FGFRs is known to be essential for survival of many cell types, although this has been shown in vertebrates to depend on the particular FGF receptor activated [73]. In *M. sexta*, when PD173074-treated animals were allowed to develop to stages later than stage 7, examination of the olfactory pathway revealed an extensive loss of NP, SZ and AN glial cells (Fig. 9B). This loss appears to be due to a combination of apoptosis (Fig. 10) and a reduction in proliferation (Fig. 9, Table 2). It is important to note that NP glial cells exhibit activated FGFRs at stage 3, before contact with ORNs (Fig. 2A), as well as in lobes chronically deprived of ORN innervation (Fig. 6E). This is consistent with other reports of a basal level of receptor tyrosine kinase (RTK) activation in the absence of ligands [74], [75] and appears necessary, in the present case, to block apoptosis. Subsequent arrival of ORN axons could then trigger additional glial responses via upregulation of FGFR activation and subsequent, developmentally relevant, activation of various downstream pathways [76]. We were not able to differentiate levels of FGFR activation at different stages by immunocytochemistry; future work will focus on questions of developmental regulation of FGFR activation and the relative localization of FGFRs to plasma membrane vs nucleus as well as possible shifts in activation of different second-messenger pathways.

### Blocking glial FGFR activation: effects on neurons

In addition to the obvious effect of FGFR inactivation on NP glial cell migration, several, more subtle, effects were noted that suggest that loss of FGFR activation disrupts the effect of glial cells on the growth patterns of axons in the sorting zone and dendrites in the developing glomeruli.

First, blockade of glial cell FGFR activation led to altered growth patterns of ORN axons as they navigated the sorting zone. Normally, ORN axons arrive at the sorting zone as a mixed population of MFas II-positive and MFas II–negative axons. On entering the sorting zone ORN axons reorient and refasciculate into MFas II-positive and MFas II–negative bundles (Fig. 1B, 11A). In PD173074-treated animals, the SZ glia had migrated outward to form a sorting zone of normal length and glial density during the early stages of axon ingrowth, yet ORN axons did not exhibit fasciculation changes as they traversed the sorting zone (Fig. 11B).

The unusual axonal phenotype is not due to reduced numbers of SZ glia, since their numbers appear to decline significantly only after most of the ORN axons have completed their traverse through the sorting zone. This suggests that, despite the normal distribution and number of the SZ glia, they were unable to induce or support a robust and early fasciculation response in ORN axons, perhaps due to reduced FGFR-dependent production of one or more glia-derived molecules.

It is interesting to note that although ORN axon fasciculation in the sorting zone appears to be perturbed or delayed, fasciculation and course changes do occur as axons leave the sorting zone, as though axons are suddenly able to respond to some targeting cue (arrowheads in Fig. 11B). In addition, ORNs that label with an antibody to Ankyrin B were seen to extend and join to form a single terminal branch cluster in approximately the correct position (Fig. 12). Thus the signaling pathways that underlie final targeting must be independent of the expression and activation of glial FGFRs.

Second, AL neuron dendrites extended beyond their normal territory into the apical region of their glomerulus, which normally is occupied predominantly by ORN axon terminals. This can not be explained by an absence of ORN axon terminals, as they were found to be present and to terminate in the usual, apical part of the glomeruli (Fig. 7). One possibility is that blocking activation of glial FGFRs prevents the glia from releasing one or more signals that limit AL neuron dendrite outgrowth. A second possibility is that PD173074 blocks a retrograde signaling mechanism in which activation of glial FGFRs and subsequent downstream events normally feed back onto ORN terminals, affecting their ability to signal to AL neurons and thereby control arborization of AL neuron dendrites. There is reason to consider this possibility, as we have shown that blockade of nitric oxide release from ORN terminals leads to a similar AL neuron dendrite overgrowth phenotype in combination with a lack of NP glial migration [15]. We noted in that report that it was necessary to block release of nitric oxide several days before the normal start of glial migration and AL neuron outgrowth, raising the possibility that nitric oxide functioned, in part, to regulate gene expression in glia and/or AL neurons, preparing them to be able to respond to additional signals from ORN axons at the appropriate time. We know, too, from γ-irradiation and hydroxyurea experiments in which glial cell proliferation was blocked, that we do not see any obvious changes in development of the architecture of the antennal lobe until approximately 75% of the glial cells are eliminated [12]–[14]. Given that the complement of glial cells is not greatly reduced in the treated antennal lobes during the period of axon ingrowth, the effects on dendritic outgrowth are not simply a consequence of a severely reduced number of glial cells.

### Possible mechanisms of FGFR activation

Vertebrate FGFRs can be activated by a large number of FGFs, and two FGFs, named Pyramus and Thisbe or FGF8-like 1&2, have been described and shown to activate Heartless in *Drosophila* embryos [77]–[80] and in imaginal eye discs of *Drosophila* larvae [55], [56]. Pyramus, in particular, is an attractive candidate for initiating the events described in the present work, as it induces glial cell proliferation and migration in the *Drosophila* eye disc [55], [56]. We have searched for Lepidopteran homologs via translated BLAST searches of Lepidopteran ESTs but have found no matches, so the question of whether classical ligands exist for the *Manduca* FGFR remains unanswered.

FGFRs and EGFRs in both vertebrates and in *Drosophila* have been shown to be activated via homophilic and heterophilic interactions in *cis* and in *trans* between the IgCAMs L1/Neuroglian, NCAM/Fasciclin II, and N-cadherin [19]–[23], [38], [39], [81], [82]. We know from our previous work [17], [18] that the transmembrane form of *Manduca* Fasciclin II (TM-Fas II) is expressed by ORN axons and Neuroglian is expressed by ORN axons and central glia. The GPI-linked isoform of MFas, GPI-Fas II, previously was detected in what appeared to be a subset of AN glia [17], but more recently has been found to be expressed by NP, SZ, and AN glia (Gibson, unpublished). These results are timely in view of recent work describing homophilic interactions between neuronal TM-Fas II and glial GPI-Fas II in *Drosophila* embryos, in which glial migration along axons is regulated by cell-surface expression of neuronal TM-Fas II [83].

In summary, we have found evidence for activated FGFRs on central and peripheral glia of the primary olfactory pathway of *Manduca sexta*. Our results indicate that these FGFRs are not required for ORN axon targeting, but are essential for 1) the proliferation and survival of glial cells, 2) the ORN-induced migration of NP glial cells to surround glomeruli, 3) the glia-regulated establishment of normal dendritic territory in the glomeruli, and 4) the glia-induced organization of ORN axon fasciculation through the sorting zone. Despite these important effects, ORN axons do manage to target at least one identified glomerulus correctly when FGFR activation is blocked, suggesting a robust targeting system with some degree of redundancy.

## Supporting Information

Figure S1 **The ATP and PD173074 binding pocket of** ***Bombyx*** **FGFR contains all of the amino acid residues shown to contact PD173074 in the human FGFR1.** The relevant regions of the highly conserved tyrosine kinase domains of human and *Bombyx* FGFRs were aligned using the Clustal-W amino acid alignment program at: http://npsa-pbil.ibcp.fr/cgi-bin/npsa_automat.pl?page=/NPSA/npsa_clustalw.html [84], [85]. The amino acid residues that contact the PD173074 molecule [36] are highlighted in yellow.
(DOC)

Figure S2 **Preadsorption of the anti-pFGFR antibody with its antigenic phospho-peptide eliminates glial labeling.** **A:** Stage-6 brains were labeled with the anti-pFGFR antibody (magenta) using the standard protocol. Syto 13 (green) was used to label nuclei. **B:** The anti-pFGFR antibody was preadsorbed with the antigenic phospho-peptide. Glial labeling was largely eliminated, as was most of the AL neuron labeling. **C:** When the anti-pFGFR antibody was omitted from the protocol (No Primary Control), weak labeling of the AL neurons was visible, suggesting that the residual labeling in **B** was partly due to non-specific labeling by the secondary antibodies. LG, MG = lateral and medial group of AL neuron cell bodies. **A′–C′:** pFGFR channel alone. Projection depths were 15 µm.
(TIF)

Figure S3 **Labeling for FGFRs and HSPG matches that for pFGFRs.** **A:** An antibody to the extracellular domain of human FGFR1 labels glial cells and AL neuron cell bodies as was seen for pFGFRs. **A′:** An enlarged and deeper region of **A** reveals labeled glial processes (arrowheads) and colocalization of labels for FGFRs and DNA (arrow). **B:** An antibody to heparan sulfate proteoglycans, necessary components of the FGF-FGFR-HSPG complexes required for ligand-mediated FGFR activation, labels glial cells and AL neuron cell bodies, but not AL neuron dendrites or ORN axons. Arrowheads point to glial processes surrounding glomeruli (*). **C–C″:** A higher magnification image of NP glia surrounding a single stage-7 glomerulus. Glial processes were labeled (arrowheads in **C, C″**) as was seen for FGFRs (**A′**). **C″:** Merged HSPG and Syto 13 images demonstrates colocalization in the nuclei, as was seen for pFGFRs (**Fig. 4** and panel **S3A′**). **D–D″:** AN glia also display labeling of processes (arrowheads) and nuclei. LG, MG = lateral and medial group of AL neuron cell bodies. Projection depths = 5 µm in **A, A′**. Images were single optical sections in **B–D″**. 40× objective in **C–D″**.
(TIF)

Figure S4 **Apoptag channel version of** **Figure 10** **.** **A–F:** Control and PD173074-treated animals were allowed to develop to various stages, then dissected and analyzed for apoptotic nuclei using the TUNEL technique. Numerous apoptotic nuclei were seen in treated animals (panels **B,D,F**), but few to no apoptotic nuclei were seen in control animals (panels **A,C,E**) at all stages examined. Arrowheads in panels **B,D** indicate apoptotic nuclei among the medial group of antennal lobe neurons. Inset in panel **F** shows a higher magnification view of a region within the sorting zone of the antennal nerve. Projection depths = 10 µm.
(TIF)

Figure S5 **ORNs exhibit no evidence for FGFRs.** **A:** Antennal nerves of untreated stage 7 females were labeled with the pFGFR antibody (magenta). Dark spaces between glia are filled with ORN axons. **B:** Antennae from the same animals were sectioned in longitudinal section and labeled with the pFGFR antibody. Counterstains (green) were Syto 13 in panel **A** to show nuclei and LEL-fitc in **B** to delineate ORN cell bodies and sensilla. Using the collection parameters from panel **A** we found no labeling of ORN cell bodies or sensillar processes (panel **B**, pFGFR channel alone in **B′**). **C:** Antennal nerves of untreated stage 7 females were labeled with both the pFGFR (**C**, magenta) and Fasciclin (**C′**, blue) antibodies. Syto 13 labeling of nuclei (green) serves to align the images in panels **C,C′**. We found no evidence of pFGFR labeling in ORN axons. Projection depths were 15 µm in **A**, 3 µm in **C**. Image in **B** was a single optical section (40× objective).
(TIF)

Figure S6 **The** ***M. sexta*** **Eph receptor is unlikely to be affected by PD173074.** Alignments of a section of the tyrosine kinase domains of the human FGFR1 and the *M. sexta* Eph receptor shows that many of the amino acids required for binding PD173074 (yellow highlighting) are different in the latter case (gray highlighting), suggesting that, as for vertebrates, PD173074 would not affect *M. sexta* Eph receptors. Note, too, the large gaps needed to achieve the alignment (compare to Figure S1).
(DOC)
