# Supplementary material for: Activation of Glial FGFRs Is Essential in Glial Migration, Proliferation, and Survival and in Glia-Neuron Signaling during Olfactory System Development
Source: PLoS One. 2012 Apr 6;7(4):e33828. doi: 10.1371/journal.pone.0033828 (PMC3320908; doi:10.1371/journal.pone.0033828)
Supplement: Figure S1 — The ATP and PD173074 binding pocket of Bombyx FGFR contains all of the amino acid residues shown to contact PD173074 in the human FGFR1. The relevant regions of the highly conserved tyrosine kinase domains of human and Bombyx FGFRs were aligned using the Clustal-W amino acid alignment program at: http://npsa-pbil.ibcp.fr/cgi-bin/npsa_automat.pl?page=/NPSA/npsa_clustalw.html [84], [85]. The amino acid residues that contact the PD173074 molecule [36] are highlighted in yellow. (DOC) [file pone.0033828.s001.doc]

Fig S1

FGFR1_Hsap LGKPLGEGCFGQVVLAEAIGLDKDKPNRVTKVAVKMLKSDATEKDLSDLISEMEMM

FGFR_Bmori LGKVLGEGEFGKVVKAECIGILK--PGLQSVVAVKMLKEGHTDAEMMALVSEMEMM

*** **** **:** **.**: * *. : *******.. *: :: *:******

FGFR1_Hsap KMIGKHKNIINLLGACTQDGPLYVIVEYASKGNLREYLQARRPPGLEYCYNPSHNP

FGFR_Bmori KMIGKHVNIINLLGCCTQDGPLYVIVEYAPNGNLREFLRNHRPGNRYESPNEDLKE

****** *******.**************.:*****:*: :** . . * . :

FGFR1_Hsap EEQLSSKDLVSCAYQVARGMEYLASKKCIHRDLAARNVLVTEDNVMKIADFGLA

FGFR_Bmori KKTLTQKDLVSFSYQVARGMEYLASRRCIHRDSAARNVLVSDDCVLKIADFGLA

:: *:.***** :************::***** *******::* *:********
