# Supplementary figures and images for: Activation of Glial FGFRs Is Essential in Glial Migration, Proliferation, and Survival and in Glia-Neuron Signaling during Olfactory System Development
Source: PLoS One. 2012 Apr 6;7(4):e33828. doi: 10.1371/journal.pone.0033828 (PMC3320908; doi:10.1371/journal.pone.0033828)

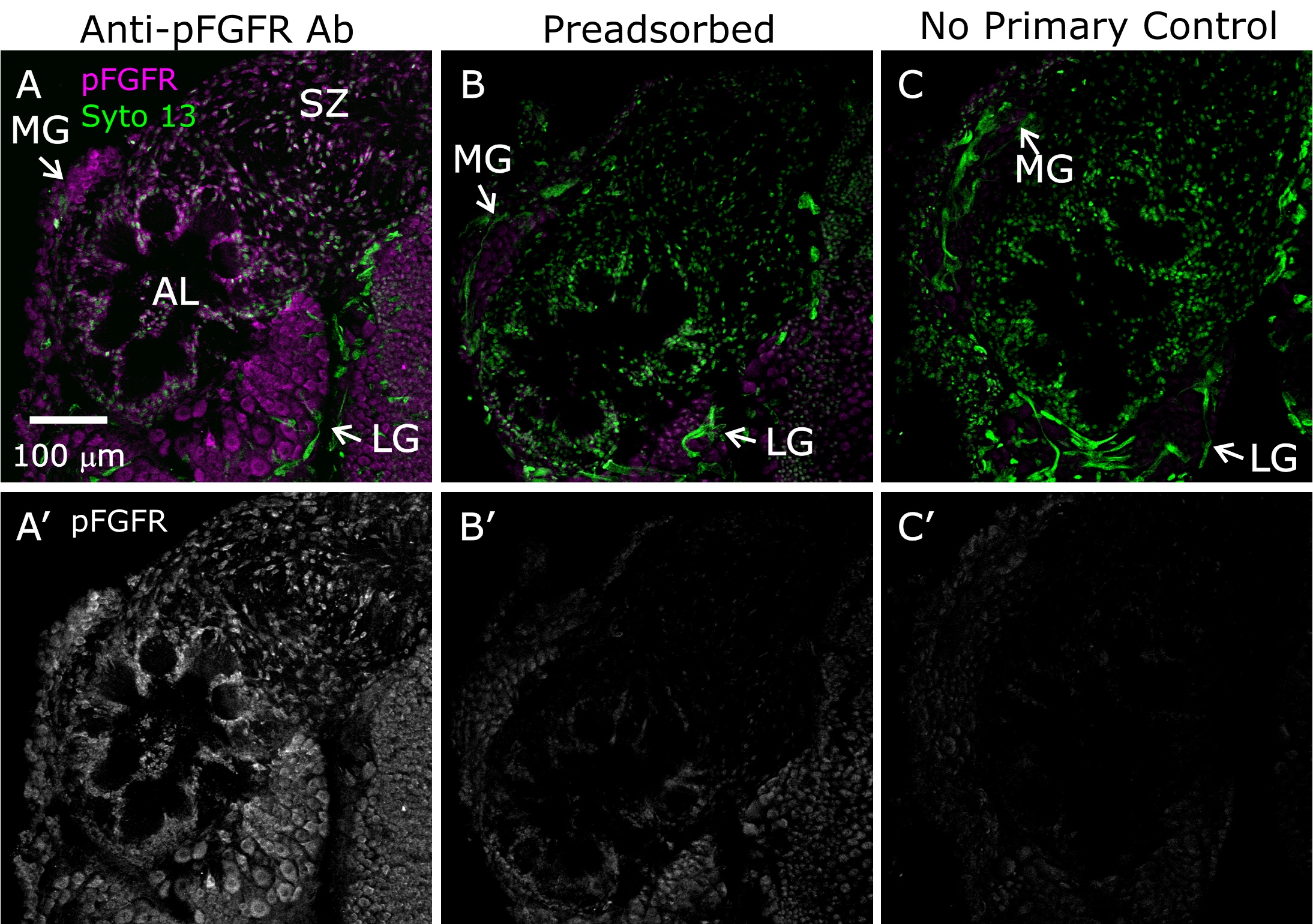

Supplement: Figure S2 — Preadsorption of the anti-pFGFR antibody with its antigenic phospho-peptide eliminates glial labeling. A: Stage-6 brains were labeled with the anti-pFGFR antibody (magenta) using the standard protocol. Syto 13 (green) was used to label nuclei. B: The anti-pFGFR antibody was preadsorbed with the antigenic phospho-peptide. Glial labeling was largely eliminated, as was most of the AL neuron labeling. C: When the anti-pFGFR antibody was omitted from the protocol (No Primary Control), weak labeling of the AL neurons was visible, suggesting that the residual labeling in B was partly due to non-specific labeling by the secondary antibodies. LG, MG = lateral and medial group of AL neuron cell bodies. A′–C′: pFGFR channel alone. Projection depths were 15 µm. (TIF) [file pone.0033828.s002.tif]

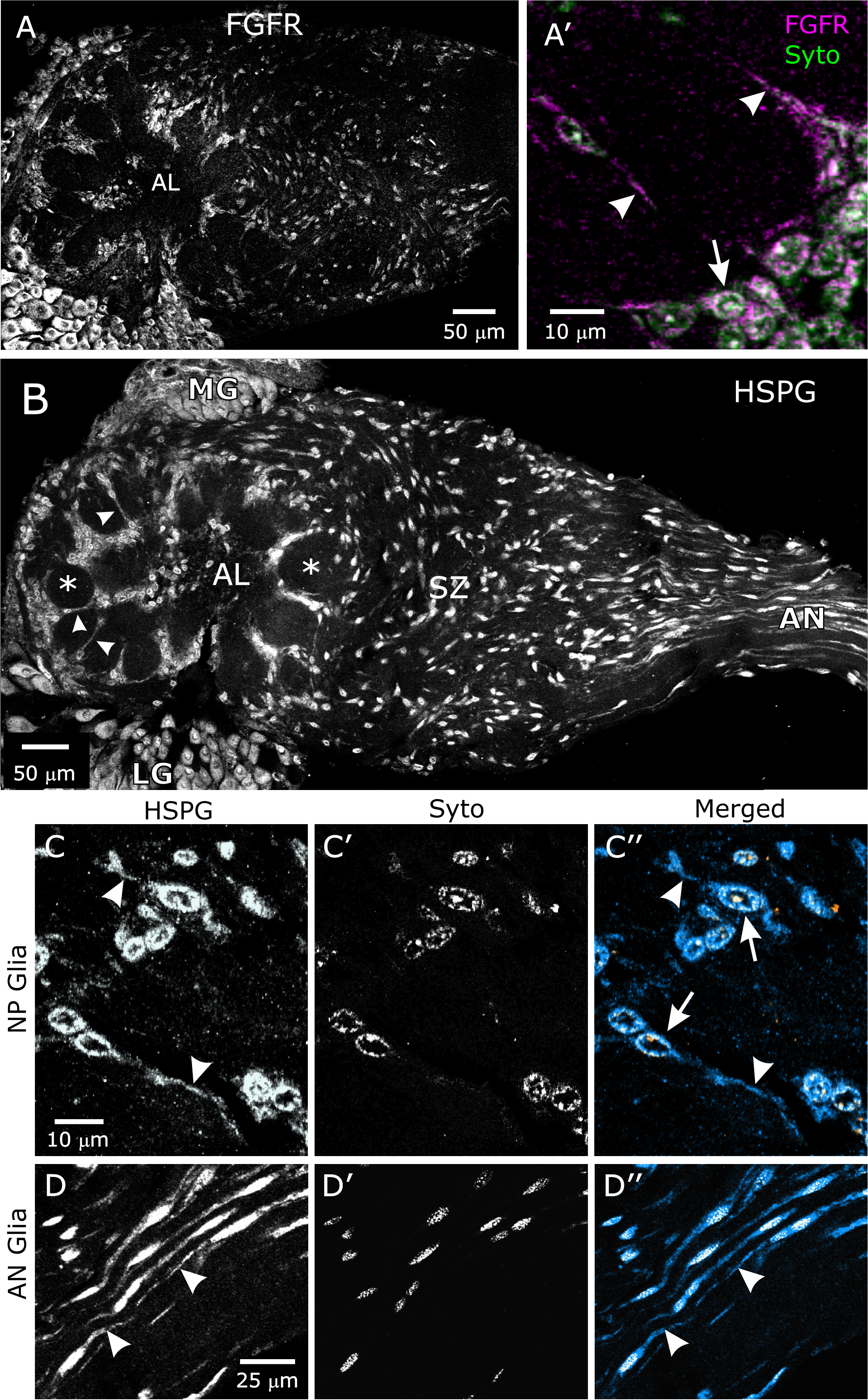

Supplement: Figure S3 — Labeling for FGFRs and HSPG matches that for pFGFRs. A: An antibody to the extracellular domain of human FGFR1 labels glial cells and AL neuron cell bodies as was seen for pFGFRs. A′: An enlarged and deeper region of A reveals labeled glial processes (arrowheads) and colocalization of labels for FGFRs and DNA (arrow). B: An antibody to heparan sulfate proteoglycans, necessary components of the FGF-FGFR-HSPG complexes required for ligand-mediated FGFR activation, labels glial cells and AL neuron cell bodies, but not AL neuron dendrites or ORN axons. Arrowheads point to glial processes surrounding glomeruli (*). C–C″: A higher magnification image of NP glia surrounding a single stage-7 glomerulus. Glial processes were labeled (arrowheads in C, C″) as was seen for FGFRs (A′). C″: Merged HSPG and Syto 13 images demonstrates colocalization in the nuclei, as was seen for pFGFRs ( Fig. 4 and panel S3A′). D–D″: AN glia also display labeling of processes (arrowheads) and nuclei. LG, MG = lateral and medial group of AL neuron cell bodies. Projection depths = 5 µm in A, A′. Images were single optical sections in B–D″. 40× objective in C–D″. (TIF) [file pone.0033828.s003.tif]

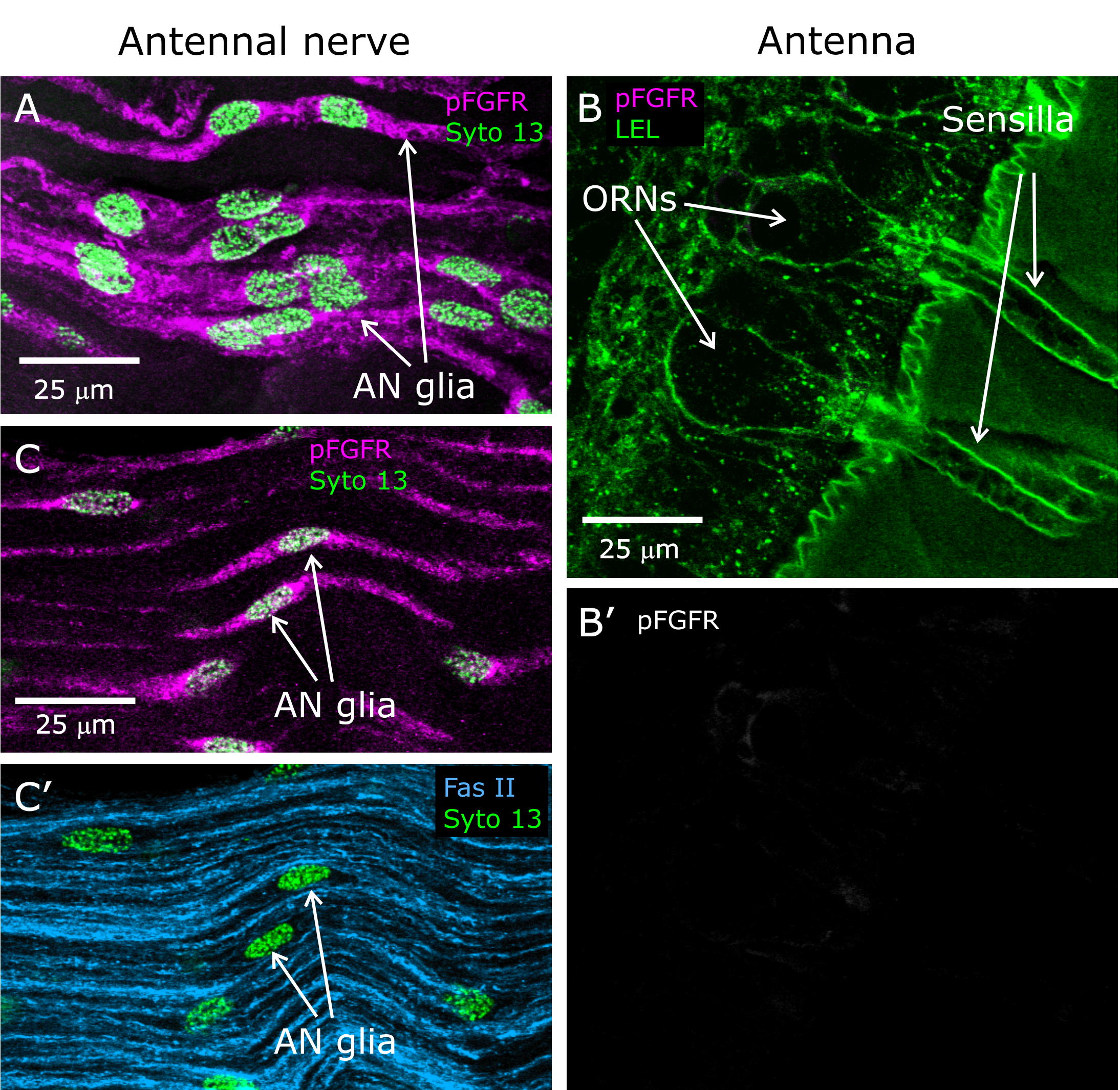

Supplement: Figure S5 — ORNs exhibit no evidence for FGFRs. A: Antennal nerves of untreated stage 7 females were labeled with the pFGFR antibody (magenta). Dark spaces between glia are filled with ORN axons. B: Antennae from the same animals were sectioned in longitudinal section and labeled with the pFGFR antibody. Counterstains (green) were Syto 13 in panel A to show nuclei and LEL-fitc in B to delineate ORN cell bodies and sensilla. Using the collection parameters from panel A we found no labeling of ORN cell bodies or sensillar processes (panel B, pFGFR channel alone in B′). C: Antennal nerves of untreated stage 7 females were labeled with both the pFGFR (C, magenta) and Fasciclin (C′, blue) antibodies. Syto 13 labeling of nuclei (green) serves to align the images in panels C,C′. We found no evidence of pFGFR labeling in ORN axons. Projection depths were 15 µm in A, 3 µm in C. Image in B was a single optical section (40× objective). (TIF) [file pone.0033828.s005.tif]
