# Supplementary material for: Activation of Glial FGFRs Is Essential in Glial Migration, Proliferation, and Survival and in Glia-Neuron Signaling during Olfactory System Development
Source: PLoS One. 2012 Apr 6;7(4):e33828. doi: 10.1371/journal.pone.0033828 (PMC3320908; doi:10.1371/journal.pone.0033828)
Supplement: Figure S6 — The M. sexta Eph receptor is unlikely to be affected by PD173074. Alignments of a section of the tyrosine kinase domains of the human FGFR1 and the M. sexta Eph receptor shows that many of the amino acids required for binding PD173074 (yellow highlighting) are different in the latter case (gray highlighting), suggesting that, as for vertebrates, PD173074 would not affect M. sexta Eph receptors. Note, too, the large gaps needed to achieve the alignment (compare to Figure S1). (DOC) [file pone.0033828.s006.doc]

Fig S5

Hs_FGFR1 LGKPLGEGCFG----------------------------------QVV--------

Ms_EphR LNYRNGEVYSGPERPAKTSSNATTPLFAGTGSRTYIDPHTYEDPNQAVREFAREID

*. ** * *.*

Hs_FGFR1 -----LAEAIGLD-----K------DKPNRVTKVAVKMLKSDATEKDLSDLISEME

Ms_EphR ASCITIEAIIGGGEFGDVCRGKLKLASCGQEIDVAIKTLKPGSTERARRDFLAEAS

: ** . . .: .**:* **..:**: *:::* .

Hs_FGFR1 MMKMIGKHKNIINLLGACTQDGPLYVIVEYASKGNLREYLQARRPPGLEYCYNPSH

Ms_EphR IMGQF-EHPNVIFLQGVVTKCNPIMIITEFMENGSLDTFLRAN-------------

:* ::* *:* * *. *: .*: :*.*: .:*.* :*:*.

Hs_FGFR1 NPEEQLSSKDLVSCAYQVARGMEYLASKKCIHRDLAARNVLVTEDNVMKIADFGLA

Ms_EphR --DGKFMVLQLVGMLRGIATGMQYLSEMNYIHRDLAARNVLVNSHLVCKIADFGLS

: :: :**. :* **:**:. : ************... * *******:
